# Rhodobacteraceae methanethiol oxidases catalyze methanethiol degradation to produce sulfane sulfur other than hydrogen sulfide

**DOI:** 10.1128/mbio.02907-23

**Published:** 2024-02-08

**Authors:** Qun Cao, Xuanyu Liu, Qingda Wang, Zongzheng Liu, Yongzhen Xia, Luying Xun, Huaiwei Liu

**Affiliations:** 1 State Key Laboratory of Microbial Technology, Shandong University, Qingdao, China; 2 Qingdao Institute of Animal Husbandry and Veterinary Medicine, Qingdao, China; 3 School of Molecular Biosciences, Washington State University, Pullman, Washington, USA; University of California, Irvine, Irvine, California, USA

**Keywords:** DMSP, Rhodobacteraceae, methanethiol, methanethiol oxidase, H_2_S, sulfane sulfur

## Abstract

Methanethiol (MT) is a sulfur-containing compound produced during dimethylsulfoniopropionate (DMSP) degradation by marine bacteria. The C–S bond of MT can be cleaved by methanethiol oxidases (MTOs) to release a sulfur atom. However, the cleaving process remains unclear, and the species of sulfur product is uncertain. It has long been assumed that MTOs produce hydrogen sulfide (H_2_S) from MT. Herein, we studied the MTOs in the Rhodobacteraceae family—whose members are important DMSP degraders ubiquitous in marine environments. We identified 57 MTOs from 1,904 Rhodobacteraceae genomes. These MTOs were grouped into two major clusters. Cluster 1 members share three conserved cysteine residues, while cluster 2 members contain one conserved cysteine residue. We examined the products of three representative MTOs both *in vitro* and *in vivo*. All of them produced sulfane sulfur other than H_2_S from MT. Their conserved cysteines are substrate-binding sites in which the MTO–S–S–CH_3_ complex is formed. This finding clarified the sulfur product of MTOs and enlightened the MTO-catalyzing process. Moreover, this study connected DMSP degradation with sulfane sulfur metabolism, filling a critical gap in the DMSP degradation pathway and representing new knowledge in the marine sulfur cycle field.

**IMPORTANCE:**

This study overthrows a long-time assumption that methanethiol oxidases (MTOs) cleave the C–S bond of methanethiol to produce both H_2_S and H_2_O_2_—the former is a strong reductant and the latter is a strong oxidant. From a chemistry viewpoint, this reaction is difficult to happen. Investigations on three representative MTOs indicated that sulfane sulfur (S^0^) was the direct product, and no H_2_O_2_ was produced. Finally, the products of MTOs were corrected to be S^0^ and H_2_O. This finding connected dimethylsulfoniopropionate (DMSP) degradation with sulfane sulfur metabolism, filling a critical gap in the DMSP degradation pathway and representing new knowledge in the marine sulfur cycle field.

## INTRODUCTION

The organosulfur compound dimethylsulfoniopropionate (DMSP) produced by marine phytoplankton, angiosperms, animals, and some heterotrophic bacteria can reach several Pg (10^15^ g) per year (1–3). DMSP provides important carbon and sulfur sources for marine microbial communities (4, 5). Three DMSP metabolic pathways, demethylation, oxidation, and cleavage, have been discovered in marine microorganisms (6–8). Among them, demethylation is the most important one because 50% to 90% of DMSP is processed through this pathway (9). Via the demethylation pathway, DMSP is cleaved into two major intermediates, acetaldehyde and methanethiol (MT). The former then enters the carbon cycle, whereas the latter has an uncertain fortune. It was reported that MT was assimilated or broken down to formaldehyde and H_2_S (10–13).

It is estimated that 1 to 1.8 Pg MT is produced from DMSP per year, and this amount does not include other MT sources, such as methylation of sulfide, degradation of sulfur-containing amino acids, and dimethyl sulfide (DMS) (14–16). Chemically, MT is a volatile compound like H_2_S and DMS, and only a few measurements of MT in the environment have been reported. Lomans et al. measured the MT of ditches in a minerotrophic peatland in the Netherlands and found that its concentration reached 3–76 nM in sediments and 1–8 nM in surface freshwater (17). In seawater, MT concentration was suggested to be 0.02–2 nM (18, 19). Microbial uptake and degradation are important sinks for MT. Radiotracer experiments showed that trace levels of MT (0.5 nM) were rapidly taken up and then utilized as carbon and sulfur sources by marine bacterioplankton (20). To be assimilated by bacteria, C–S bond of MT is first cleaved by methanethiol oxidase (MTO), but the cleavage process and sulfur products are uncertain (12, 21–23).

MTO was distributed in many species, including bacteria, archaea, humans, fish, birds, and plants (24–26). It was proposed that the human MTO can degrade MT into H_2_S, hydrogen peroxide (H_2_O_2_), and formaldehyde (HCHO) (27). Philipp et al. once used a bacterial recombinant l-methionine gamma-lyase to produce MT *in situ* and then used enterocyte MTO to treat the product. During this coupled enzyme assay, H_2_S and H_2_O_2_ were detected (28). In addition, it was observed that MTO-containing bacteria can produce H_2_S when they consume MT (26). It has long been assumed that in marine microorganisms, DMSP-derived MT was first degraded to H_2_S by MTO, which was further oxidized to sulfane sulfur (S^0^) by sulfide:quinone oxidoreductase (SQR) or directly used for cysteine synthesis.

*Ruegeria pomeroyi* DSS-3 is a model bacterium of the Rhodobacteraceae family. It is also the first sequenced heterotrophic marine bacterium and has been widely used in the study of marine sulfur metabolism. Previous studies indicate that this strain contains a well-known DMSP demethylation pathway, and the sequence analysis indicates that it contains a proposed MTO (12, 29). Therefore, it is a good candidate bacterium used in MTO study. Herein, we found out that its MTO (hereafter, we renamed it as RpMTO) catalyzed MT degradation to produce sulfane sulfur other than H_2_S. Two MTOs from other strains showed the same activity. Our findings clarified the sulfur product of the MTO-catalyzed MT degradation reaction and filled a critical gap in the DMSP degradation pathway.

## RESULTS

### RpMTO degraded MT but produced no H_2_O_2_

**Fig 1:**
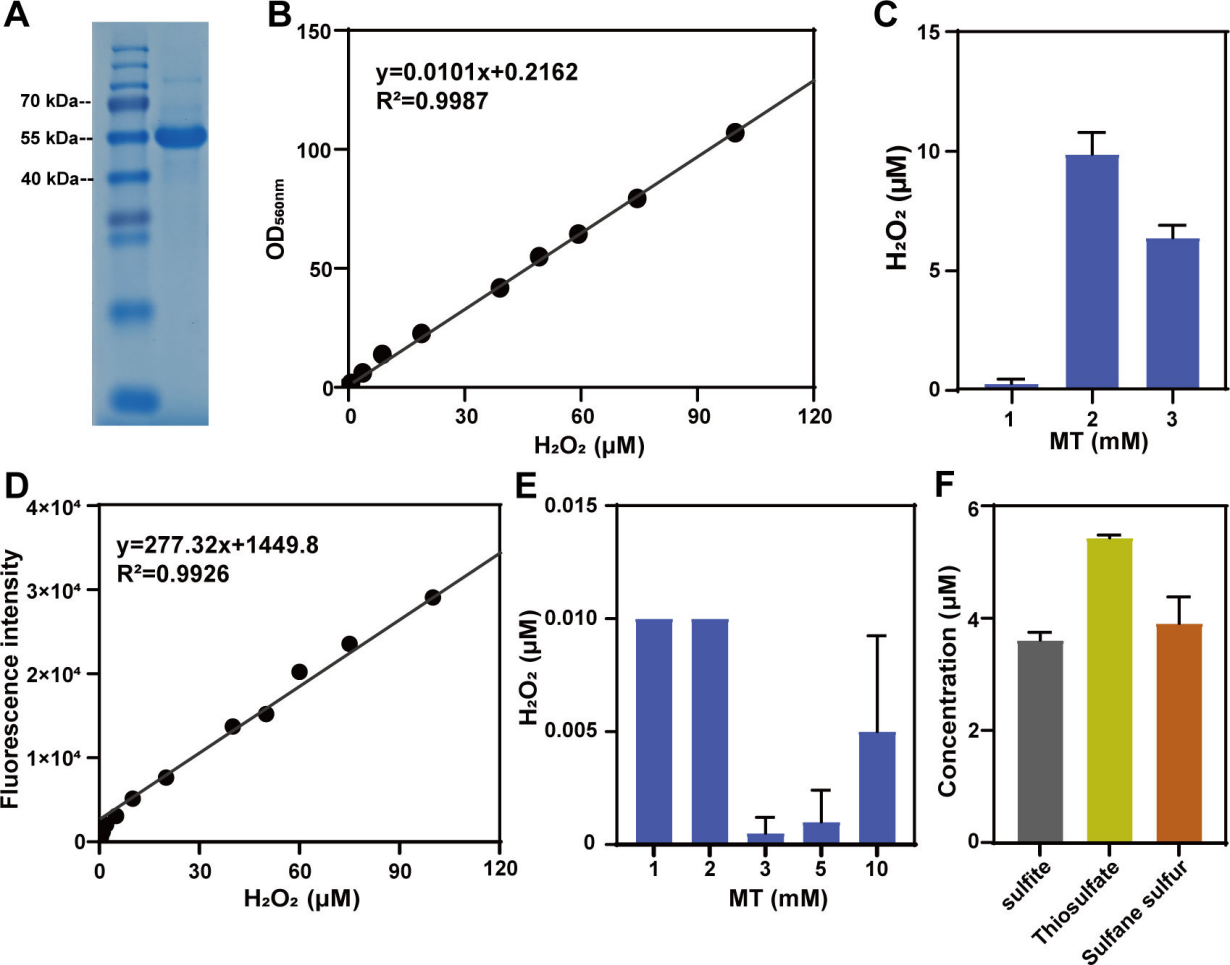
Characterization of RpMTO and analysis of its products. (**A**) SDS-PAGE analysis of the purified RpMTO. The left lane is a weight marker, and the right lane is RpMTO. (**B**) The standard calculation curve of the Peroxide Assay Kit method made in this study. (**C**) Using Peroxide Assay Kit to analyze the produced H_2_O_2_ from enzymatic reaction. (**D**) The standard calculation curve of the horseradish peroxidase (HRP)-catalyzed 10-acetyl-3,7-dihydroxyphenox-azine (ADHP) method made in this study. (**E**) Using HRP-catalyzed ADHP to analyze the produced H_2_O_2_ from enzymatic reaction. (**F**) Products from the chemical reaction of H_2_S with H_2_O_2_. High-performance liquid chromatograph (HPLC) spectra of them are provided in Fig. S1. HSSH, hydrogen persulfide.

Marine bacterium *R. pomeroyi* DSS-3 degrades DMSP to produce MT via the demethylation pathway. It also contains an RpMTO (WP_011242048.1) encoded by SPOA0269, suggesting that it can also degrade MT. We cloned the RpMTO-encoding gene and expressed it in *Escherichia coli* BL21 (DE3). The expressed RpMTO (fused with an N-terminal His-Tag) was purified using a nickel column. During cell disruption and protein purification processes, no dithiothreitol (DTT) or other reductants were added. The SDS-PAGE analysis indicated that its molecular weight was around 55 kDa (Fig. 1A), near the calculated molecular weight (50.4 kDa).

Freshly purified enzyme was subjected to activity analysis. According to the assumed activity, RpMTO can degrade MT in the presence of oxygen to produce HCHO, H_2_S, and H_2_O_2_ (reaction 1).


(1) $$CH_{3}SH+O_{2}+H_{2}O\to HCHO+H_{2}S+H_{2}O_{2}$$

To test its activity, we mixed purified RpMTO with MT. The gas chromatography-mass spectrometry (GC–MS) analysis indicated that 77 µM MT was oxidized by RpMTO. We then tried to quantify the produced H_2_O_2_. A commercial H_2_O_2_ assay kit was used. According to our test, the detection limit of this kit was about 10 µM (Fig. 1B). However, in the above-mentioned reacting conditions, no H_2_O_2_ product was detected from the RpMTO-catalyzing reaction by this kit. We then increased the MT concentration to 1–3 mM. Still, only around 10 µM H_2_O_2_ or less was detected (Fig. 1C). Considering that both the proteins RpMTO and MT have weak absorbance at 560 nm, and 10 µM was on the edge of the kit’s detection limit, the detected H_2_O_2_ concentration cannot be trusted.

We then used the H_2_O_2_ fluorescence probe 10-acetyl-3,7-dihydroxyphenox-azine (ADHP) for detection. According to our test, this probe can detect as low as 0.5 µM H_2_O_2_ (Fig. 1D). To avoid potential disturbance, RpMTO was precipitated down by acetonitrile and removed by centrifugation after the reaction. Only about 0.005 µM H_2_O_2_ was detected even when MT was increased to 10 mM (Fig. 1E). Therefore, the detected H_2_O_2_ concentration with the ADHP probe cannot be trusted either.

It is possible that the produced H_2_O_2_ quickly reacted with H_2_S and was completely consumed in reaction 2 (unbalanced).


(2) $$H_{2}S+H_{2}O_{2}\to SO_{3}^{2-}+S_{2}O_{3}^{2-}+HSSH$$

To test this possibility, we mixed equal amounts of H_2_O_2_ and H_2_S. The produced sulfur-containing compounds were derivatized with monobromobimane (mBBr) and subjected to HPLC analysis. Sulfite, thiosulfate, and hydrogen persulfide (HSSH) were detected (Fig. 1F; Fig. S1). However, when using the same method to analyze the products of the RpMTO-catalyzing reaction, no sulfite or thiosulfate was detected. These results suggested that reaction 2 was not involved in the process of RpMTO-catalyzed MT degradation. Combining the above results, we concluded that no H_2_O_2_ was produced by RpMTO; that is, RpMTO did not catalyze reaction 1.

### RpMTO produced sulfane sulfur rather than H_2_S from MT

**Fig 2:**
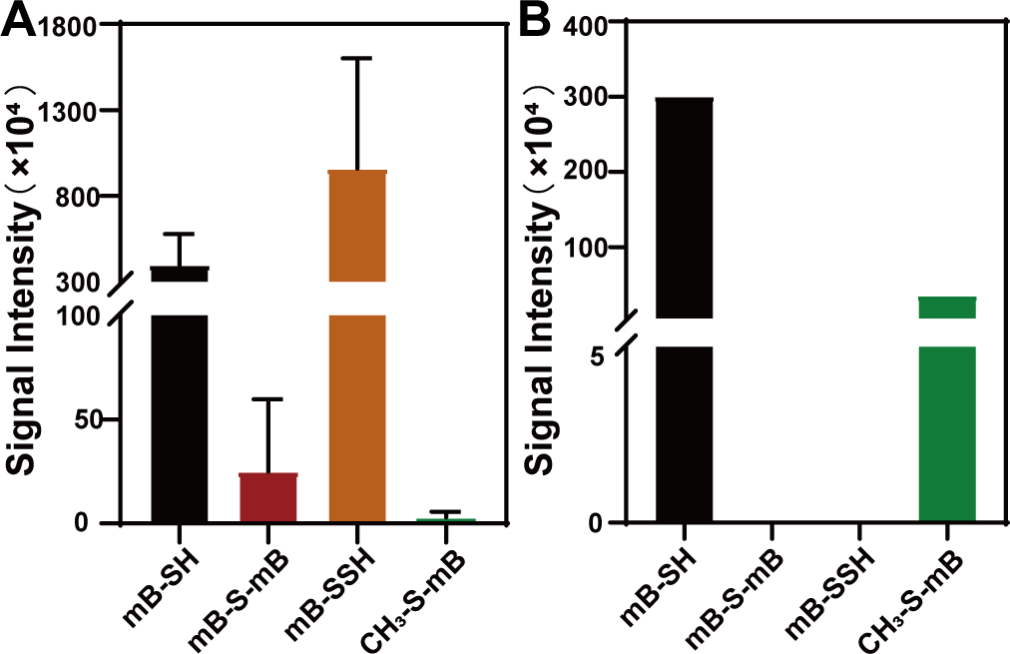
LC–ESI-MS analysis of the sulfur products produced by RpMTO. (**A**) MS signal intensities of sulfur products from RpMTO-catalyzed MT degradation. mB–SH and mB–S–mB are H_2_S derivatives (by mBBr); mB–SSH is an HSSH derivative, and CH_3_–S–mB is an MT derivative. (**B**) The MS signal intensities of sulfur products from the control experiment (no RpMTO). MS spectra of them are provided in Fig. S4.

To examine the sulfur-containing products generated from the RpMTO-catalyzing reaction, purified RpMTO was mixed with MT. The products were derivatized with mBBr and analyzed by liquid chromatography-electrospray ionization-mass spectrometry (LC–ESI-MS). Again, no sulfite or thiosulfate derivative was detected. H_2_S derivatives mB–SH and mB–S–mB were present. The signal intensity of the former was 4.0 × 10^6^ and the latter was 2.5 × 10^5^ (Fig. 2A; Fig. S2). Surprisingly, the derivative of HSSH (mB–SSH) was also present, and its signal intensity was 9.6 × 10^6^ (Fig. S3), which was much higher than that of H_2_S derivatives. The remaining MT can be derivatized by mBBr to form CH_3_–S–mB. This derivative compound also was present with a signal intensity of 3 × 10^4^ (Fig. S4).

As the control, we also diluted MT in reaction buffer without RpMTO and then derivatized with mBBr. The LC–ESI-MS analysis indicated that except for CH_3_–S–mB, mB–SH also was present with a signal intensity of 3 × 10^6^ (Fig. 2B). No mB–S–mB was present. These results were unexpected because we did not know why and how mBBr reacted with methanethiol to generate mB–SH but not mB–S–mB. Nonetheless, the critical finding was that no HSSH derivative mB–SSH was present, indicating that sulfane sulfur species HSSH was only produced from the RpMTO-catalyzing reaction; however, H_2_S was not produced.

To examine whether the amount of the produced sulfane sulfur was equal to another product HCHO, we quantified both products in the enzymatic reaction system. The amount of sulfane sulfur atom (quantified as the total sulfane sulfur by the cyanide method) was 20.0 ± 4.3 µM, while that of HCHO was 24.9 ± 1.9 µM. The ratio was 0.8:1, a little less than 1:1, probably because a portion of the produced sulfane sulfur became H_2_S.

In 1987, Suylen et al. once detected sulfane sulfur from *Hyphomicrobium* sp. strain EG MTO catalyzed MT oxidation (22). They proposed that the product was from reaction 3.


(3) $$H_{2}S+O_{2}\to S^{0}+H_{2}O_{2}$$

However, when we incubated the H_2_S solution at the aerobic condition for 30 min, no sulfane sulfur production was detected. In addition, reaction 3 is against our observations of H_2_S-related experiments, from which we found that H_2_S was not readily oxidized by oxygen without the help of related enzymes/oxidants. Therefore, we concluded that the sulfane sulfur detected from the RpMTO-catalyzing reaction was not from reaction 3.

### RpMTO produced sulfane sulfur *in vivo*

We used three *R. pomeroyi* DSS-3 mutants to further verify the sulfur product of RpMTO *in vivo*, the first one is ∆*pdo*, in which the persulfide dioxygenase-encoding gene *pdo* was deleted. Therefore, this strain loses sulfane sulfur oxidation activity and can accumulate sulfane sulfur inside cells. The second one is ∆*sqr*∆*fccAB*, in which the H_2_S oxidation enzyme-encoding genes *sqr* and *fccAB* were deleted. Therefore, this strain loses H_2_S oxidation activity and can release H_2_S into culture once it is produced intracellularly (H_2_S easily passes through the cell membrane). The third one is ∆*mtoX*, in which the RpMTO-encoding gene was deleted. This strain was used as a negative control.

**Fig 3:**
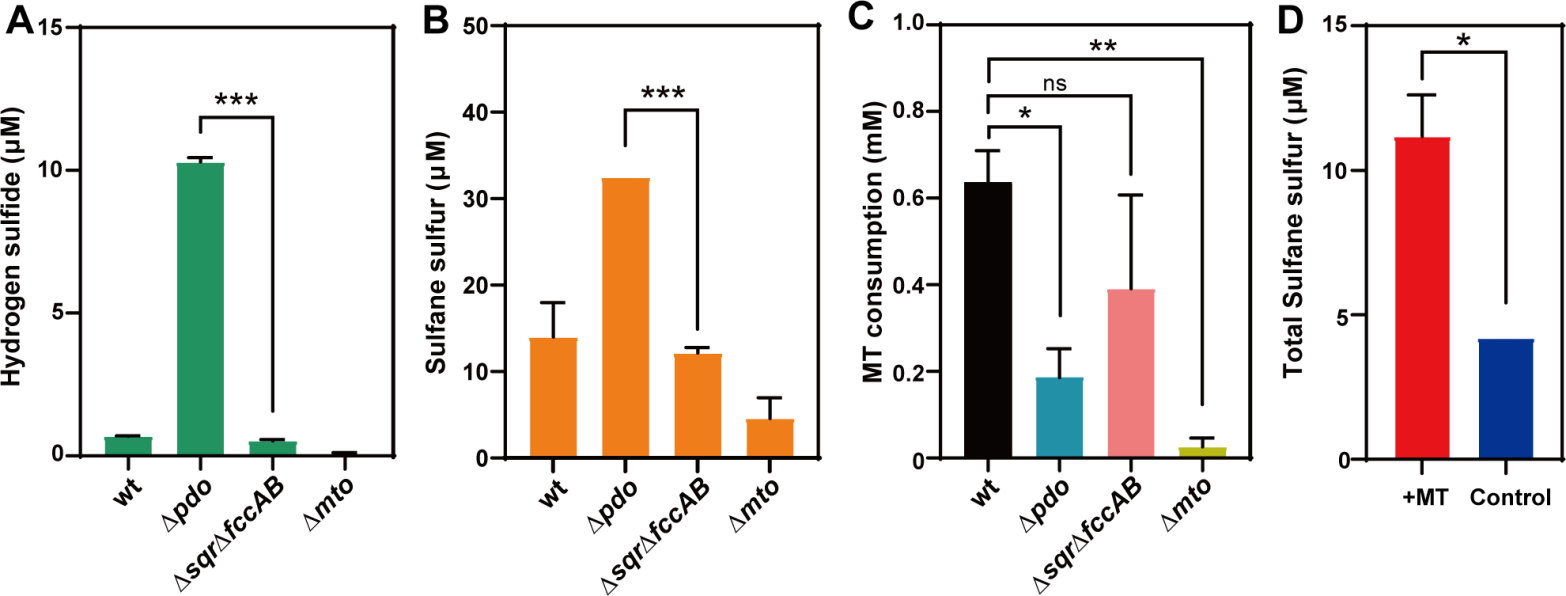
MT degradation and product analysis of RpMTO-containing strains. (**A**) Hydrogen sulfide production from *R. pomeroyi* DSS-3 wt and mutants. (**B**) Sulfane sulfur production from *R. pomeroyi* DSS-3 wt and mutants. (**C**) MT consumed by *R. pomeroyi* DSS-3 wt and mutants. (**D**) Sulfane sulfur production from *E. coli* BL21(DE3) containing the plasmid pET28a-RpMTO. *t*-tests were performed to calculate the *P*-values, and asterisks indicate statistically significant differences (^∗^*P* < 0.05, ^∗∗^*P* < 0.01, ^∗∗∗^*P* < 0.001). ns, no significant difference.

We mixed cells of *R. pomeroyi* DSS-3 wild type (wt) and three mutants with MT individually. After 2 h incubation, H_2_S in the cell-MT culture and sulfane sulfur in cells were quantified (H_2_S is cell membrane permeable but sulfane sulfur is not). The latter was quantified as the total sulfane sulfur using an HPLC-based method (30). Theoretically, if H_2_S is the direct product of RpMTO, it will accumulate in ∆*sqr*∆*fccAB* culture but not in ∆*pdo* culture. The results showed that, on the contrary, ∆*sqr*∆*fccAB* culture did not accumulate more H_2_S than the other three cultures (Fig. 3A). ∆*pdo* culture was the one that accumulated the highest concentration of H_2_S. Previous studies have indicated that a high concentration of intracellular sulfane sulfur is toxic to cells. To decrease the sulfane sulfur level, cells either oxidize sulfane sulfur to less toxic sulfite or reduce it to releasable H_2_S via glutathione (GSH) or enzyme-mediated reactions. For strains that have no persulfide dioxygenase (PDO) (such as *E. coli* and yeast), they cannot conduct the oxidation reaction, and hence, reduction is the only choice (30, 31). This should be the reason why Δ*pdo* culture accumulated more H_2_S than the others. Its H_2_S was from the reduction of the RpMTO-produced sulfane sulfur.

On the other hand, if sulfane sulfur is the direct product of RpMTO, sulfane sulfur will accumulate in ∆*pdo* cells but not in ∆*sqr*∆*fccAB* cells because PDO in the latter is still active and can oxidize the produced sulfane sulfur to sulfite. The results, indeed, showed that ∆*pdo* cells accumulated significantly higher amounts of intracellular sulfane sulfur than ∆*sqr*∆*fccAB* cells did (Fig. 3B).

Theoretically, if the PDO-mediated sulfane sulfur oxidation pathway is impeded, the RpMTO-catalyzed MT degradation should be affected due to the accumulation of sulfane sulfur, which is toxic to cells at high concentrations (30, 31). To check this, we used GC–MS to analyze the MT degradation capabilities of wt and three mutants. The wt metabolized 0.53 ± 0.16 mM MT, and ∆*sqr*∆*fccAB* metabolized 0.39 ± 0.15 mM MT. In comparison, ∆*pdo* metabolized 0.18 ± 0.05 mM MT (Fig. 3C). These results indicated that blocking the sulfane sulfur oxidation pathway in *R. pomeroyi* DSS-3, indeed, severely impeded its MT degradation capability, whereas blocking H_2_S oxidation pathway just had mild influence. In addition, ∆*mtoX* lost most of the MT degradation activity (metabolized only 0.03 ± 0.02 mM MT), and it produced almost no H_2_S and the lowest amount of sulfane sulfur (Fig. 3A through C). These results indicated that RpMTO was the critical enzyme for MT degradation in *R. pomeroyi* DSS-3.

*E. coli* BL21(DE3) contains no H_2_S or sulfane sulfur oxidation enzymes (no SQR, flavocytochrone c sulfide dehydrogenase (FCC), or PDO), which makes it a clean background for testing RpMTO activity. We constructed a pET28a-RpMTO plasmid and transformed it into *E. coli* BL21(DE3). The strain was induced by isopropyl β-D-1-thiogalactopyranoside (IPTG), and then 10 mL cell suspension (OD_600_ = 3.0) was collected. We mixed MT with this cell suspension. After 1 h incubation, both H_2_S and sulfane sulfur were quantified. Cell suspension without MT addition was used as a control. H_2_S was not produced by the experimental group (cells mixed with MT) or control, and the experimental group produced more intracellular sulfane sulfur than the control did (Fig. 3D). The above results confirmed that sulfane sulfur, other than H_2_S, was the product of RpMTO *in vivo*.

### The cysteine residue Cys28 was required for RpMTO activity

**Fig 4:**
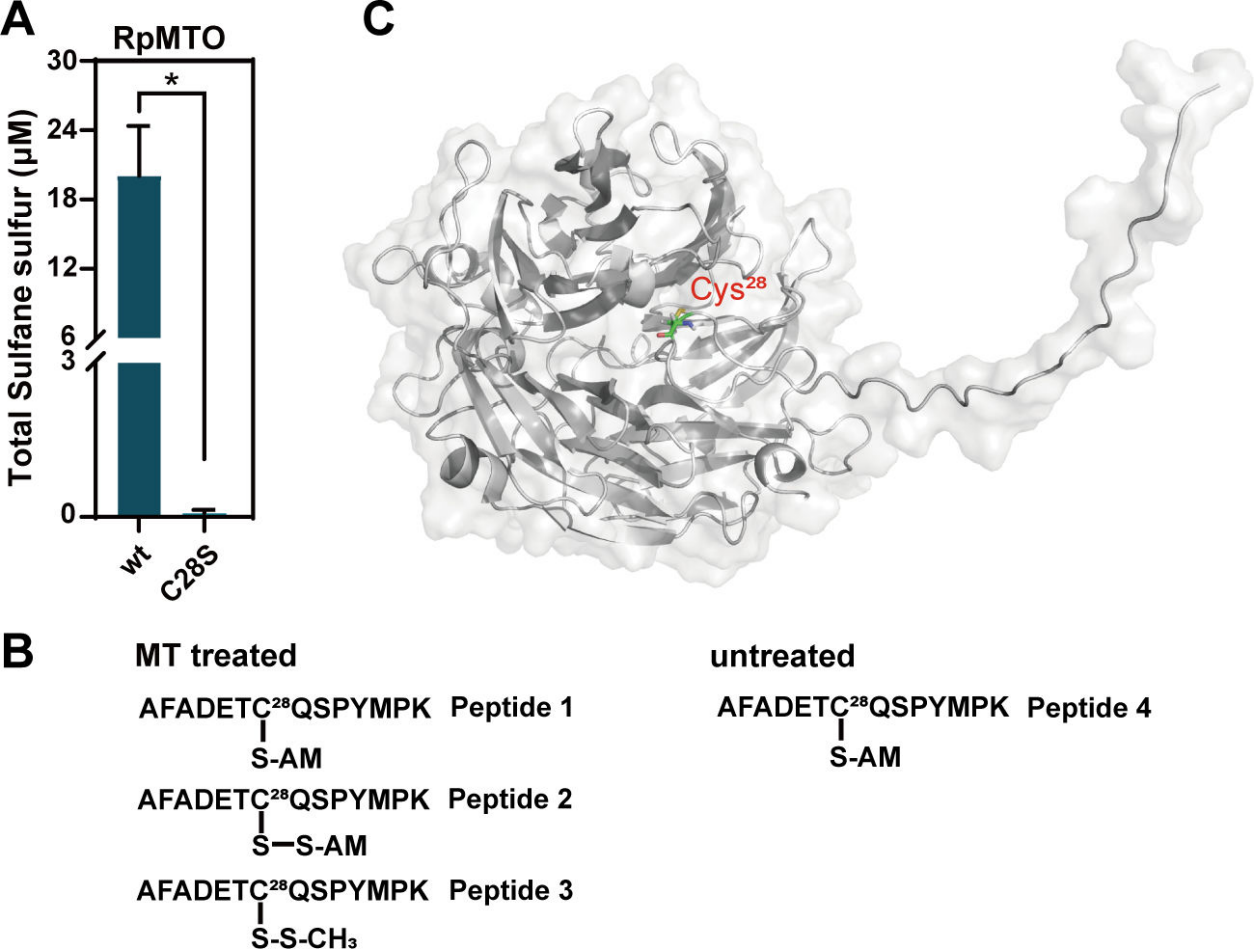
Characterization of RpMTO mutant, its peptide, and modeled structure. (**A**) RpMTO_C28S_ mutant lost sulfane sulfur-producing activity. (**B**) LC–MS/MS analysis of MT-treated and untreated RpMTO. MS^2^ spectra of the peptides are provided in Fig. S8. (**C**) Modeled structure of RpMTO using AlphaFold 2. The conserved cysteine residue (Cys28) is shown as sticks, ∗, *P* < 0.05.

The other enzymes that catalyze C–S bond breakage, such as 3-mercaptopyruvate sulfurtransferase (3-MST) and cystathionine β-synthase (CBS), require cysteine residues for their activities (32, 33). To check whether RpMTO is the same case, we mutated the only cysteine of RpMTO (Cys28) to serine. The mutant RpMTO_C28S_ was expressed in *E. coli* and purified using a nickel column. We used the sulfane sulfur-producing ability to judge its activity. After mixing purified RpMTO_C28S_ with MT, sulfane sulfur production was barely detected, suggesting that Cys28 was required for MT degradation activity (Fig. 4A).

To check how RpMTO reacts with MT, purified RpMTO was incubated with MT. The MT treated-RpMTO was labeled with iodoacetamide (IAM) and then subjected to trypsin digestion, followed by LC–MS/MS analysis. RpMTO without MT treatment was used as a control. For the MT-treated RpMTO, three Cys28-containing peptides were identified (Fig. 4B). In peptide 1, Cys28 residue was directly blocked by IAM to form Cys_28_–S–AM (+57.02 Da) (Fig. S5). In peptide 2, a mass increase of 88.99 Da on Cys28 residue was identified (Fig. S6), suggesting that Cys_28_–S–S–H was blocked by IAM to form Cys_28_–S–S–AM. In peptide 3, a mass increase of 45.99 Da on Cys28 residue was identified, corresponding to Cys_28_–S–S–CH_3_ modification (Fig. S7). For the MT-untreated RpMTO control, only a peptide with Cys_28_–S–AM modification was identified (+57.02 Da, peptide 4), corresponding to the direct blockage of Cys28 residue by IAM (Fig. S8). These results suggested that RpMTO used its Cys28 residue to bind MT to form a Cys_28_–S–S–CH_3_ complex.

The 3D structure of RpMTO was modeled using AlphaFold 2. The modeled structure shows that Cys28 is located in an incompact, cave-like position formed by random coils (Fig. 4C). This position is near the RpMTO surface, which makes Cys28 accessible to MT. Therefore, MT should first enter this position and then react with Cys28.

### Two types of MTO are present in Rhodobacterales

**Fig 5:**
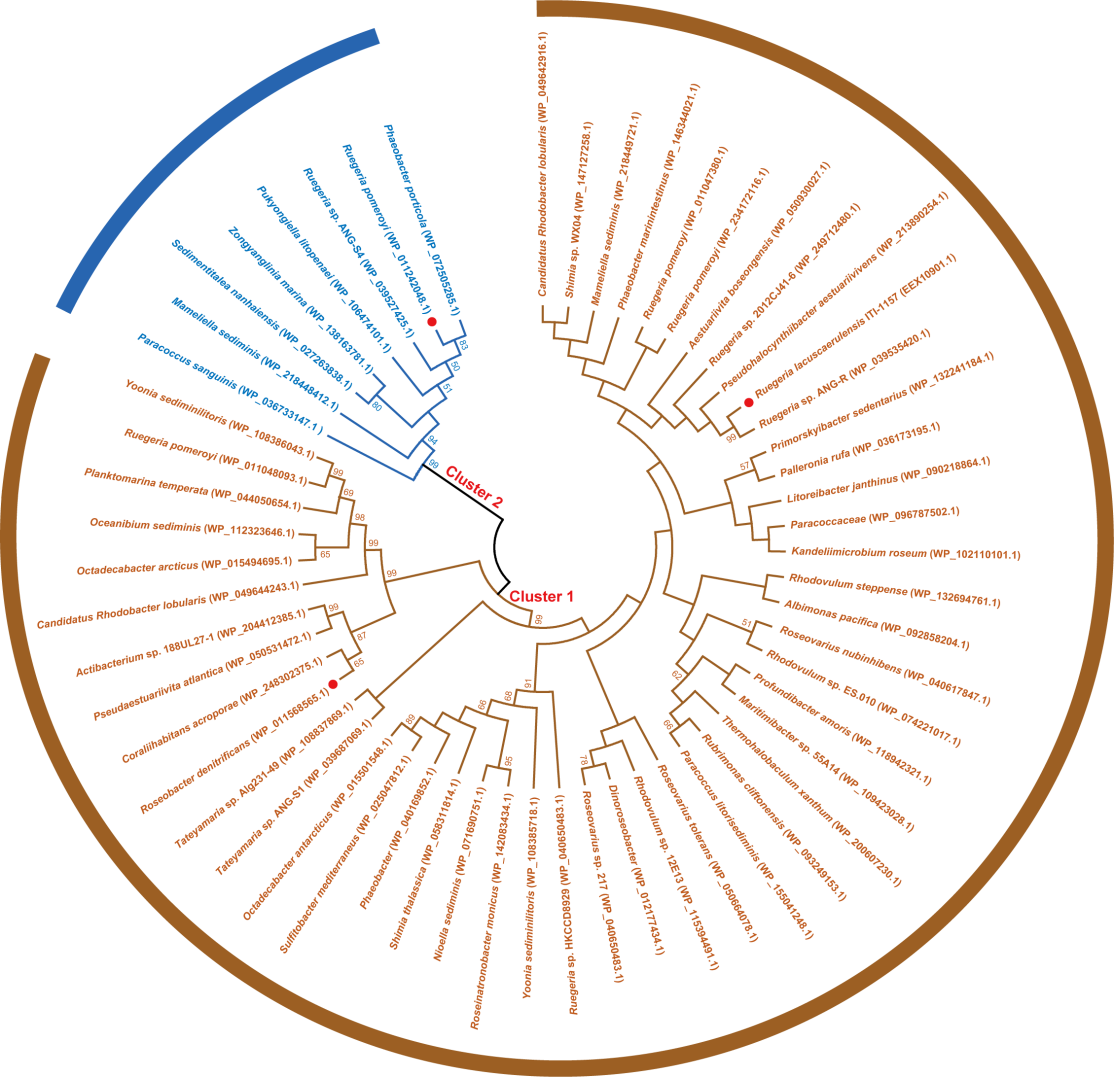
Phylogenetic analysis of 57 MTO candidates obtained from Rhodobacteraceae family. Three representative MTOs (RpMTO, RdMTO, and RlMTO) are highlighted with red dots.

Using *R. pomeroyi* DSS-3 RpMTO as the query to search homologs from Rhodobacterales genomes, we identified 57 MTOs. Most of them are predicted selenium-binding proteins. The phylogenetic tree shows that they can be grouped into two major clusters (Fig. 5). Cluster 1 contains 49 members, which are further grouped into two branches. Cluster 2 contains eight members including *R. pomeroyi* DSS-3 RpMTO.

**Fig 6:**
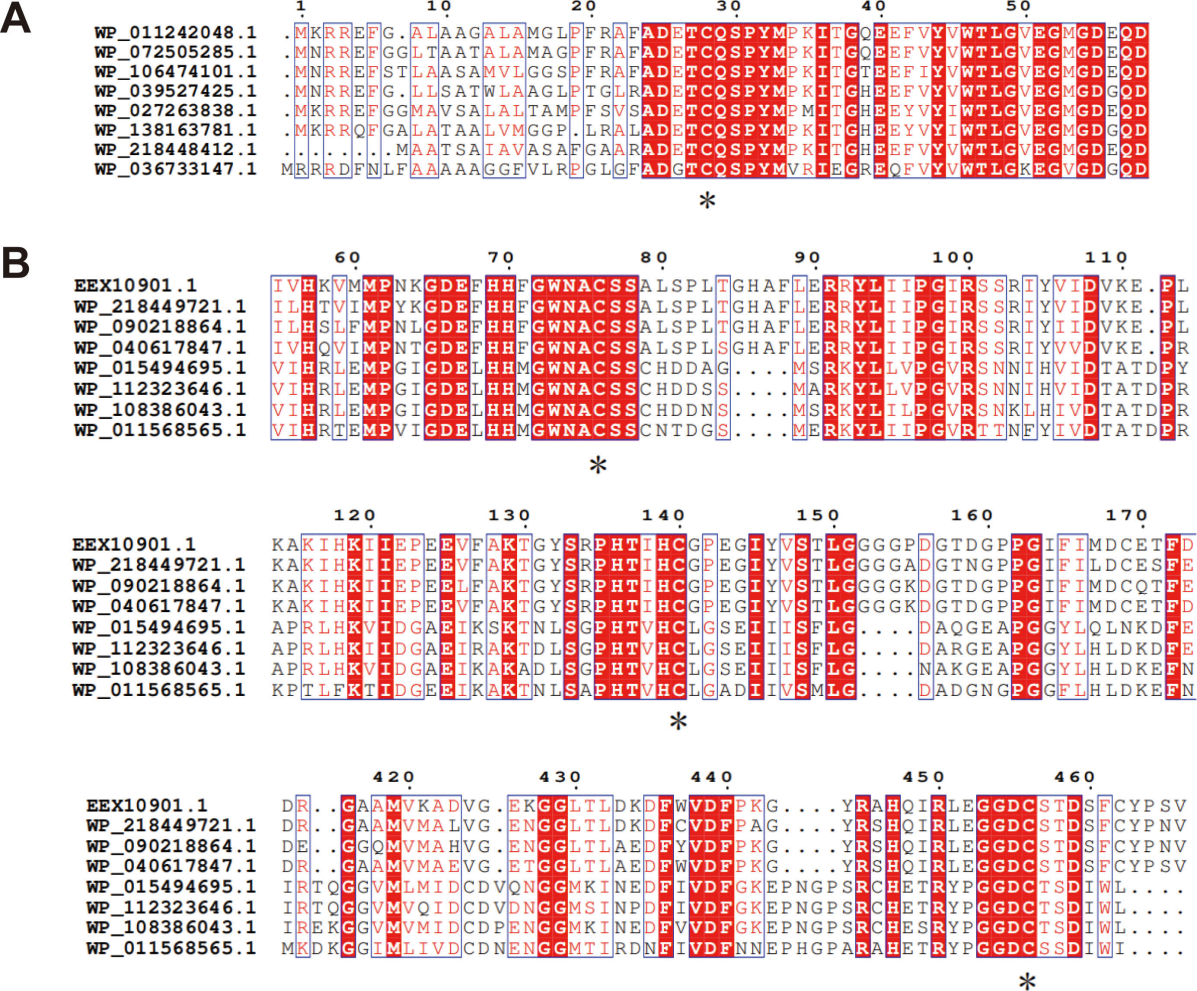
Amino acid sequence alignment of cluster 1 (**A**) and 2 (**B**) members. The conserved cysteine residues are marked with an asterisk. For cluster 2, not all members are shown here. Full sequence alignments of full members of both clusters are provided in Fig. S9 and S10.

Similar to *R. pomeroyi* DSS-3 RpMTO, the other seven members of cluster 2 also contain only one cysteine residue. We aligned their protein sequences and found that this cysteine residue conserves in all of them (Fig. 6A; Fig. S9). In addition, the neighboring residues also conserve as a “TCQSPYM” sequence. These analyses suggest that all cluster 2 MTOs catalyze MT degradation via the same mechanism.

Different from cluster 2 MTOs, cluster 1 MTOs contain multiple cysteine residues. Protein sequence alignment indicates that there are three conserved cysteine residues in most of them, located in three conserved sequences “GWNACS,” “HTV(I)HC,” and “GGDCS(T),” respectively (Fig. 6B; Fig. S10).

### Cluster 1 MTOs also produced sulfane sulfur from MT

**Fig 7:**
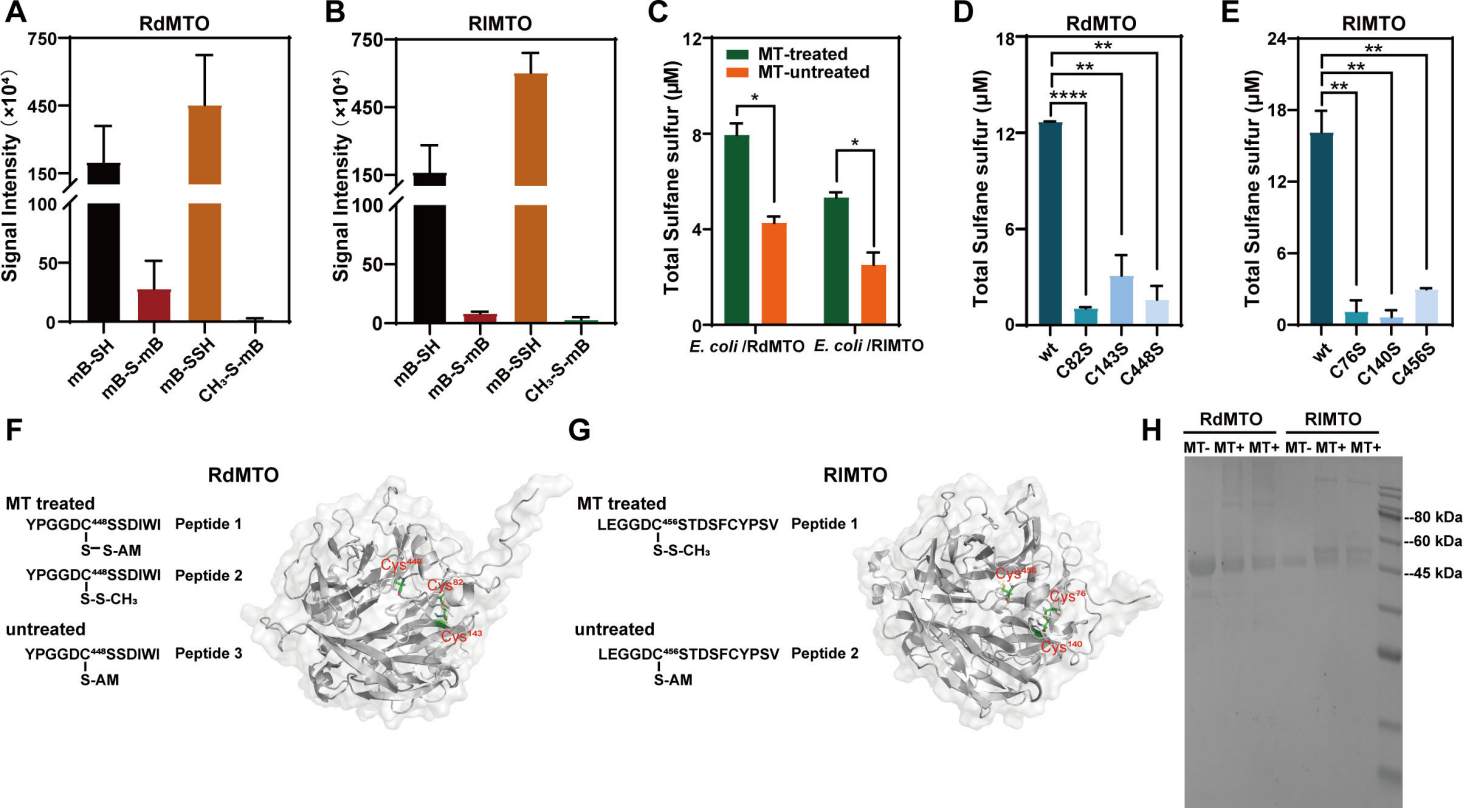
Analysis of RdMTO and RlMTO activities. (**A**) MS signal intensities of sulfur products from RdMTO-catalyzed MT degradation. (**B**) MS signal intensities of sulfur products from RlMTO-catalyzed MT degradation. (**C**) Sulfane sulfur production from *E. coli* BL21 (DE3) containing pET28a-RdMTO or pET28a-RlMTO. (**D**) Compared with RdMTO (wt), its three cysteine-to-serine mutants showed lower sulfane sulfur-producing activity. (**E**) Compared with RlMTO wt, the three cysteine-to-serine mutants showed lower sulfane sulfur-producing activity. (**F**) LC–MS/MS analysis of MT-treated and MT-untreated RdMTO. The RdMTO 3D structure modeled by AlphaFold 2. MS^2^ data of the peptides are provided in Fig. S11 to S13. (**G**) LC–MS/MS analysis of MT-treated and MT-untreated RlMTO. The RlMTO 3D structure modeled by AlphaFold 2. MS^2^ data of the peptides are provided in Fig. S14 and S15. (**I**) Non-reduced SDS-PAGE analysis of the MT-treated (MT+) and MT-untreated (MT−) RdMTO and RlMTO. For panels **C and D**, *t*-tests were performed to calculate the *P*-values, and asterisks indicate statistically significant differences (^∗^*P* < 0.05, ^∗∗^*P* < 0.01, ^∗∗∗∗^*P* < 0.0001).

*Roseobacter denitrificans* OCh114 is one of the most studied bacteria of the Roseobacter lineage (34). *Ruegeria lacuscaerulensis* ITI_1157 contains genes responsible for DMSP decomposition to produce MT (35, 36). They both have hypothetical MTOs (WP_011568565.1 and EEX10901.1, respectively). We chose their MTOs as representatives of cluster 1. MTOs of *R. denitrificans* OCh114 (renamed as RdMTO) and *R. lacuscaerulensis* ITI_1157 (renamed as RlMTO) were expressed in *E. coli* BL21(DE3) and purified with nickel columns. After mixing purified MTOs with MT, GC–MS analysis was performed to analyze MT degradation. RdMTO and RlMTO consumed 44 and 111 µM MT, respectively. The LC–ESI-MS analysis indicated that the signal intensity of H_2_S derivatives (mB–SH and mB–S–mB) from RdMTO were 1.9 × 10^6^ and 2.8 × 10^5^, respectively, whereas the signal intensity of the HSSH derivative (mB–SSH) was 4.5 × 10^6^ (Fig. 7A). The signal intensity of the two H_2_S derivatives from RlMTO were 1.6 × 10^6^ and 0.8 × 10^5^, respectively, whereas the signal intensity of HSSH derivative was 6.0 × 10^6^. For both enzymes, they produced more HSSH than H_2_S (Fig. 7B). In addition, as RpMTO, they did not produce H_2_O_2_.

To test their activities *in vivo*, we used *E. coli* BL21(DE3) harboring pET28a-RdMTO or pET28a-RlMTO plasmids. The strains were induced by IPTG. Cell suspensions (OD_600_ = 3.0) were collected and mixed with MT. Cell suspensions without MT addition were used as controls. Again, no H_2_S production was detected, and both RdMTO and RlMTO expressing *E. coli* cells produced more intracellular sulfane sulfur than controls did (Fig. 7C), indicating that RdMTO and RlMTO also catalyzed MT degradation to produce sulfane sulfur *in vivo*.

### Conserved cysteine residues were required for MT degradation for cluster 1

The three conserved cysteine residues in RdMTO and RlMTO were mutated to serine residues individually. The six mutants, RdMTO_C82S_, RdMTO_C143S_, RdMTO_C448S_, RlMTO_C76S_, RlMTO_C140S_, and RlMTO_C456S_, were expressed in *E. coli* and purified using nickel columns. As the RpMTO_C28S_ mutant, RdMTO and RlMTO mutants also lost most of the sulfane sulfur-producing activity (Fig. 7D through E), indicating that all their conserved cysteine residues were required for the MT degradation activity.

Purified RdMTO and RlMTO were incubated with MT. The MT-treated enzymes were labeled with IAM and subjected to trypsin digestion, followed by LC–MS/MS analysis. MT-untreated enzymes were used as controls. For MT-treated RdMTO and RlMTO, a common feature was that Cys–S–S–CH_3_ modification was observed in their third conserved cysteine residues (RdMTO_C448_ and RlMTO _C456_) (Fig. 7G and H; Fig. S11 to S15). No such modification was observed in MT-untreated enzymes. These results suggested that these enzymes used the third conserved cysteine residues to react with MT.

3D structures of RdMTO and RlMTO were also modeled using AlphaFold 2. Similar to the case of RpMTO, active cysteine residues of both RdMTO (Cys448) and RlMTO (Cys456) are located in incompact, cave-like positions. A difference is that their caves are looser and larger than that of RpMTO. The other two cysteines are located in very compact positions surrounded by β-sheets (Fig. 7G and H).

Non-reduced SDS-PAGE analysis indicated that freshly purified RdMTO and RlMTO were both monomers. After mixing with MT, a portion of them became dimers or tetramers (Fig. 7I). The other two cysteine residues not involved in MT binding might be involved in this process. To test this hypothesis, we performed a non-reduced SDS-PAGE analysis with RdMTO and its Cys-to-Ser mutants. RdMTO mutants containing C143S mutation all lost the dimer formation capability (Fig. S16), implying that Cys143 plays a critical role in the dimer formation process.

## DISCUSSION

The Rhodobacteraceae family is important DMSP degrader in marine environments. To achieve the final degradation of DMSP, its C–S bond needs to be cleaved to release the sulfur atom. This process is catalyzed by MTOs. The mechanism of MTO functions and the produced sulfur species remain uncertain. In this work, we studied the MTOs of the Rhodobacteraceae family. We identified 57 MTOs from 1,904 Rhodobacteraceae genomes. These MTOs were grouped into two major clusters. We examined the products of three representative MTOs (RpMTO, RdMTO, and RlMTO) both *in vitro* and *in vivo*. All of them produced sulfane sulfur other than H_2_S from MT. This finding is different from previous reports (21, 22, 37). We also found that MTO-conserved cysteines are substrate-binding sites in which the MTO–S–S–CH_3_ complex is formed. Our study clarified the product of MTO and enlightened the MTO-catalyzing process, filling a critical gap in the DMSP degradation pathway.

H_2_S has been recognized as an enzyme-mediated, intracellularly produced, sulfur-metabolizing intermediate for a long time. As for sulfane sulfur, realizing that it is commonly present inside cells and much more abundant than H_2_S just happened in the recent decade (38, 39). Intracellular sulfane sulfur exists in different forms, including Cys–S_n_H, GS_n_H, and HS_n_H (*n* ≥ 2). They are very reactive and easily reduced to H_2_S by reducing powers like GSH. Therefore, sulfane sulfur and H_2_S often coexist. The former was ignored for a long time because its detection was difficult, but new methods were quickly developed in recent decades (40, 41). Accompanied by the methodology development, some enzymes, such as 3-MST and CBS that previously were thought to be H_2_S-producing enzymes, are now considered sulfane sulfur-producing enzymes (42, 43). Actually, the finding that MTOs produce sulfane sulfur other than H_2_S is not surprising because, in previous reports of MTO-related studies (25, 27), no sulfane sulfur-detecting experiment was conducted. In 2018, Eyice et al. studied the catalyzing kinetics of RpMTO and analyzed its MT consumption and formaldehyde production, but they did not analyze its sulfur products either (12). In addition, during the enzyme purification process, reductants like DTT were often added, which can reduce sulfane sulfur to H_2_S, just like GSH does inside cells. This easily led to the wrong conclusion that H_2_S was the direct product of MTOs. In our experiments, we avoided adding DTT during MTO purification and only used freshly purified proteins for enzymatic analysis. This should be the key reason why we detected sulfane sulfur production.

**Fig 8:**
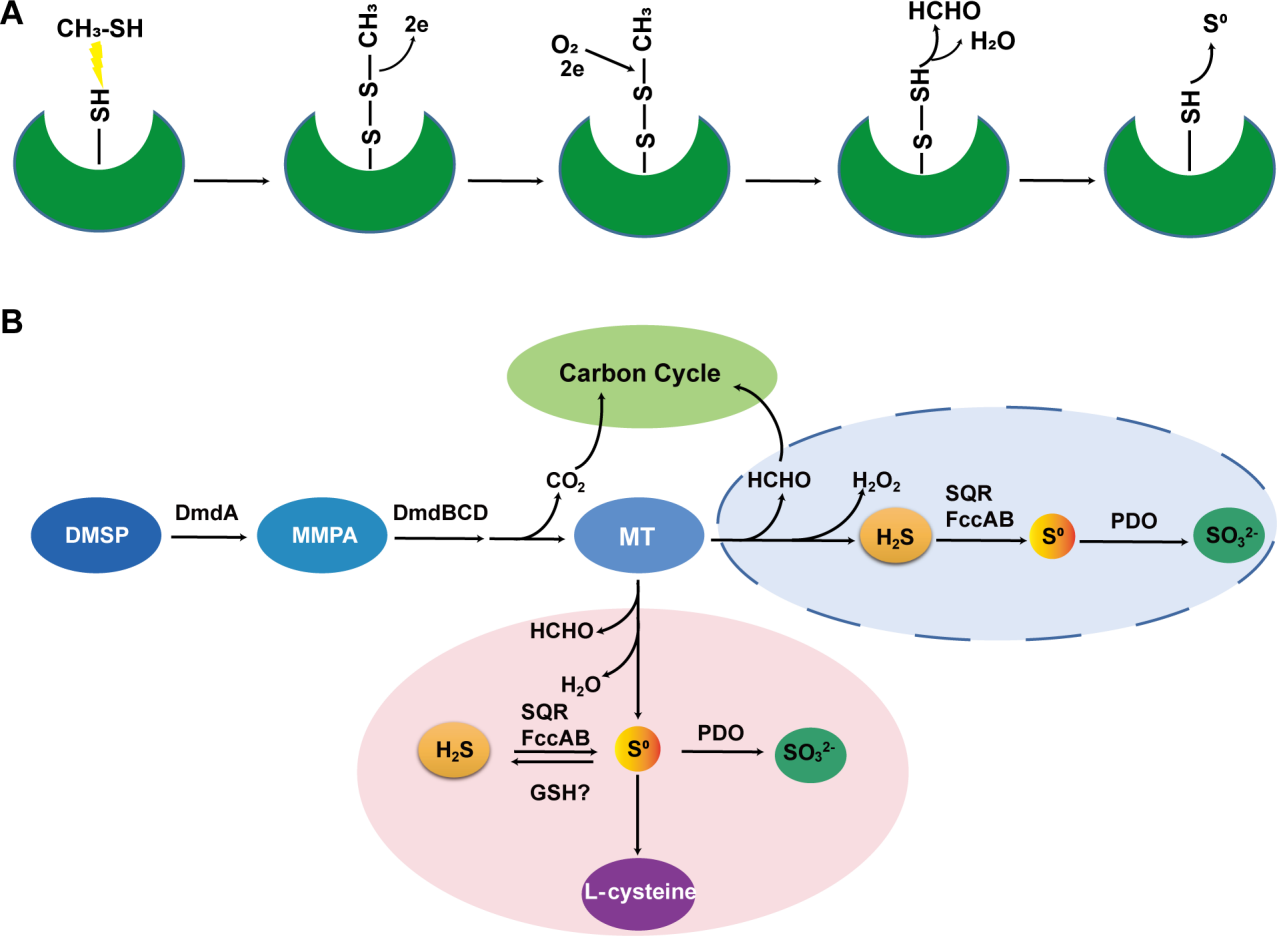
A proposed mechanism to explain how RpMTO catalyzes MT degradation to produce sulfane sulfur (**A**) and the connection of DMSP degradation with sulfur metabolism (**B**). DMSP, dimethylsulfoniopropionate; MMPA, methylmercaptopropionate.


Protein LC–MS/MS analysis indicated that the conserved cysteine residues in “GGDCS(T)” of cluster 1 MTO and in “TCQSPYM” of cluster 2 MTO are the catalyzing sites. We proposed that MT first reacts with conserved cysteine to form Cys–S–S–CH_3_ additive, and then O_2_ attacks this additive to break its C–S bond to produce HCHO and Cys–S–S–H. Since RSSH disulfide is inherently unstable (44, 45), Cys–S–S–H easily releases a sulfane sulfur atom and turns back to Cys–SH (Fig. 8A). Other amino acids spatially adjacent to the conserved cysteine residue may help in the O_2_ attack and S^0^ detachment processes. Finally, since no H_2_O_2_ production was detected, we proposed that the MTO-catalyzing reaction can be rewritten to reaction 4.

(4) $$CH_{3}SH+O_{2}\to HCHO+S^{0}+H_{2}O$$

According to the modeled structures of three MTOs, their catalyzing cysteine residues are all located in cave-like positions formed by random coils. Differently, the other two cysteines of RdMTO and RlMTO are located in a compact position surrounded by β-sheets. Therefore, MT may be accessible only to catalyzing cysteine. In consistent with our results, LC–MS/MS analysis indicated that Cys–S–S–CH_3_ additive only formed in catalyzing cysteine. However, mutating the two non-catalyzing cysteines also leads to impaired activity, demonstrating that they are not dispensable. Non-reduced SDS-PAGE analysis indicates that they are needed for dimer or tetramer formation. However, the current study of MTO is short, and whether these two cysteines are located in the oligomer formation domain is still unknown. In addition, RpMTO has a high sequence similarity (56.67%) with MTO of *Hyphomicrobium* sp. VS. The latter contains copper ions according to Eyice et al. (12). RpMTO also has conserved Trp223 and Trp386, which are supposed to be involved in the formation of a tryptophan tryptophylquinone (TTQ) cofactor. It also has His88/89, His141, and His424, which are putative copper ligands close to the TTQ (Fig. S17). Therefore, its catalyzing mechanism should be the same as the MTO of *Hyphomicrobium* sp. VS.

In conclusion, MT is an important intermediate connecting the metabolisms of organic sulfur and inorganic sulfur. In this work, we found that MTOs degraded MT, and the direct product was sulfane sulfur rather than H_2_S. This finding patches an important gap in the whole DMSP degradation pathway and correctly connects DMSP degradation with sulfane sulfur metabolism (Fig. 8B). Previously, it was thought that the sulfur atom in DMSP became H_2_S and then entered H_2_S metabolic pathway (mostly via SQR oxidation). Now, we change the route to the sulfane sulfur metabolic pathway (no SQR involved). The correction makes DMSP not only a sulfur source but also a sulfane sulfur donor, suggesting that DMSP may be involved in many regulation functions as other sulfane sulfur donors commonly do (46, 47).

## MATERIALS AND METHODS

### Strains and cultivation conditions

*R. pomeroyi* DSS-3, *R. denitrificans,* and *R. lacuscaerulensis* are gifts from Prof. Yuzhong Zhang of the Ocean University of China. *R. pomeroyi* DSS-3 derivatives ∆*pdo* and ∆*sqr∆fccAB* were constructed previously in our lab (48). *R. pomeroyi* DSS-3 ∆*mtoX* was constructed using previously reported methods (49, 50). Details of the construction method are provided in the supplemental material. *E. coli* strains used for plasmid construction and protein expression, plasmids constructed in this study are all listed in Table S1. *R. pomeroyi* DSS-3 strain was cultured in the 1/2 YTSS medium, which contains 4 g/L yeast extract, 2.5 g/L tryptone, and 20 g/L sea salts. For cultivation, *R. pomeroyi* DSS-3 strains were cultivated at 30°C with shaking (220 rpm). *E. coli* strains were cultured in lysogeny broth (LB) medium at 37°C.

Sodium hydrosulfide (NaHS) was purchased from Sigma-Aldrich (Saint Louis, MO). Hydrogen Peroxide Assay Kit was purchased from Beyotime Biotechnology (Shanghai, China). MT was purchased from Macklin Biochemical Co., Ltd (Shanghai, China), and ADHP and horseradish peroxidase (HRP) enzymes were purchased from Aladdin Biotech (Shanghai, China).

### Protein expression and purification

RpMTO-encoding gene was amplified from the genomic DNA of *R. pomeroyi* DSS-3. RdMTO- and RlMTO-encoding genes were amplified from *R. denitrificans* and *R. lacuscaerulensis* genomic DNA, respectively. Their cysteine-to-serine mutants were constructed using the QuickChange method (51). Primers used for gene expression and mutation are listed in Table S2. For MTO expression and purification in *E. coli*, a His-tag was fused to their N-terminus, and pET28a plasmid was used. More details of the expression and purification experiments are provided in the supplemental material.

### Enzymatic activity assay

MT was dissolved in propanediol (1.8 M). The enzymatic reaction was performed in 0.3 mL reaction buffer (50 mM Tris-HCl, pH 8.4) in a 1.5 mL scale tube. In the reaction buffer, purified MTO (0.3 mg/mL) was mixed with 0.6–3 mM MT. The reaction was performed at 30°C for 30 min. As a control, boiled enzyme (inactive) was also mixed with MT in the same reaction system. After the reaction, the produced H_2_O_2_ was quantified using a Hydrogen Peroxide Assay Kit or HRP-catalyzed ADHP method. The remaining MT was quantified by GC–MS. The amount of enzyme-degraded MT was calculated by subtracting MT in control (deemed as vaporized amount) from MT in the reaction system. For sulfur species determination, the products in the reaction system were derivatized with mBBr and then subjected to LC–ESI-MS analysis. The total concentration of the produced sulfane sulfur was quantified with the cyanide method (52). The produced formaldehyde was quantified using a previously reported method.

Details of H_2_O_2_, MT, sulfur species, and formaldehyde quantification are provided in the supplemental material.

### Chemical reaction and product analysis

Reaction of H_2_S with H_2_O_2_ was performed as reported previously (53). Briefly, 50  µM H_2_S was added to 50 µM H_2_O_2_ in deoxygenated Tris-HCl buffer (50 mM, pH 8.4). The reaction was conducted at room temperature for 30 min. Products were labeled with mBBr and quantified by HPLC following the protocol (54). The reaction of H_2_S with oxygen and product analysis was performed following the same protocol as the H_2_S reaction with H_2_O_2_, except that no H_2_O_2_ was added, and the reaction solution was not deoxygenated. Oxygen dissolved in the reaction solution was deemed as the reactant.

### Analysis of MTO products *in vivo*

The *R. pomeroyi* DSS-3 strains were cultured in 1/2 YTSS medium overnight. The overnight culture (1 mL) was transferred into 100 mL of fresh medium and cultured to OD_600_ = 2.0. Cells were collected by centrifugation (4,000 × *g*, 5 min) and re-suspended in Tris-HCl buffer (pH 8.4, 50 mM, 20 mM MgCl_2_, 20 g/L NaCl) to make OD_600_ = 5.0. Three *E. coli* BL21(DE3) strains harboring pET28a-RpMTO, pET28a-RdMTO, or pET28a-RlMTO plasmid were incubated in LB medium containing kanamycin (50 µg/mL). When OD_600_ reached 0.6, 0.4–0.6 mM IPTG was added, and the temperature was decreased to 25°C. After 16 h cultivation, cells were harvested by centrifugation and then re-suspended in Tris-HCl buffer to make OD_600_ = 3.0.

MT (1 mM) was added to 10 mL cell suspension in a 50 mL scale tube. The tube was then incubated at 30°C for 2 h with shaking (200 rpm). To quantify the produced H_2_S, 50 µL supernatant was taken after centrifugation. The supernatant was derivatized with mBBr and then quantified by HPLC following a previously reported protocol (54). Sulfane sulfur quantification was performed using an HPLC-based method reported previously (39). Briefly, *R. pomeroyi* DSS-3 and *E. coli* cells (treated with 1 mM MT) were collected and re-suspended in 100 µL reaction buffer (50 mM Tris-HCl, pH 9.5, 1% Triton X-100, 50 µM DTPA, and 0.5 mM sulfite) and incubated at 95°C for 10 min to convert intracellular sulfane sulfur atom into thiosulfate. The produced thiosulfate was labeled with mBBr and quantified by HPLC. The obtained sulfane sulfur amount was deemed as total sulfane sulfur.

### LC–MS/MS analysis of MTO

Purified MTO (0.3 mg/mL) was mixed with 0.6 mM MT in Tris-HCl buffer (pH 8.4, 50 mM). After incubating the mixture at 30°C for 30 min, the denaturing buffer (0.5 M Tris-HCl, 2.75 mM EDTA, 6 M guanadine-HCl, and pH 8.1) with excess iodoacetamide (0.5 M) was added to denaturalize MTO and block free thiols. LC–MS/MS analysis was performed following a previously reported protocol (55, 56). More details are provided in the supplemental material.

### SDS-PAGE and non-reduced SDS-PAGE analysis

For SDS-PAGE analysis, the purified protein was added to 5× loading buffer (CWBIO, Beijing, China) in a ratio of 5:1. After incubating the mixture at 95°C for 10 min, the sample was subjected to gel electrophoresis. For non-reduced SDS-PAGE, the loading buffer did not contain DTT. After electrophoresis, the gel was stained for 5–10 min and then placed in FluorChem Q (Thermo Fisher, Waltham, MA, USA) for imaging. To investigate how MT affects MTO status, purified MTO was divided into two portions, and the control portion was not treated with MT. The reaction portion was reacted with 1 mM MT at 30°C for 30 min. The denaturing buffer (0.5 M Tris-HCl, 2.75 mM EDTA, 6 M guanadine-HCl, and pH 8.1) with excess IAM (0.5 M) was added to denaturalize protein and block free thiols. Finally, a non-reduced SDS-PAGE analysis was performed.

### MTO structure modeling

The AlphaFold 2 algorithm (57) was used to model the tertiary structure of three MTOs. This method used the custom multiple sequence alignment option and was accessed via the Colab server on GitHub (https://github.com/sokrypton/ColabFold). The structural models of MTOs were analyzed and visualized with PyMOL (Version 1.5.0.3).

### Bioinformatic analysis

Rhodobacterales genomes were downloaded from the NCBI database (1904, update to 6 June 2022), and redundancy was removed using the CD-HIT tool. MTO candidate genes (a total of 57) in Rhodobacterales genomes were obtained by searching the database with the standalone BLASTP algorithm using conventional criteria (*E* value of ≤1e^−5^, coverage of  ≥45%, and identity of ≥25%). A phylogenetic tree was constructed by a neighbor-joining method using MEGA 7.0 with a partial deletion, p-distance distribution, and bootstrap at 1,000 repeats. Multiple sequence alignment was performed using Clustal Omega.

### Statistical information

Data of Fig. 1C, Fig. 1E, Fig. 1F, Fig. 2C, Fig. 3A through D, Fig. 4A, and Fig. 7C through E were obtained from three independent replicates and shown as average ± SD.

## SUPPLEMENTAL MATERIAL

Supplemental material mbio.02907-23-s0001.pdf Methods, Tables S1 and S2, and Figures S1-S17.

ASM does not own the copyrights to Supplemental Material that may be linked to, or accessed through, an article. The authors have granted ASM a non-exclusive, world-wide license to publish the Supplemental Material files. Please contact the corresponding author directly for reuse.
