# Supplementary material for: Rhodobacteraceae methanethiol oxidases catalyze methanethiol degradation to produce sulfane sulfur other than hydrogen sulfide
Source: mBio. 2024 Feb 8;15(3):e02907-23. doi: 10.1128/mbio.02907-23 (PMC10936201; doi:10.1128/mbio.02907-23)
Supplement: Supplemental material — Methods, Tables S1 and S2, and Figures S1-S17. [file mbio.02907-23-s0001.pdf]

Supplemental Material

Rhodobacteraceae methanethiol oxidases catalyze methanethiol degradation to produce sulfane sulfur other than hydrogen sulfide

Qun Cao[1], Xuanyu Liu[1], Qingda Wang[1], Zongzheng Liu[2], Yongzhen Xia[1], Luying Xun[1,3], Huaiwei Liu[*]

[1]State Key Laboratory of Microbial Technology, Shandong University, Qingdao, 266200, PR China.

[2]Qingdao Institute of Animal Husbandry and Veterinary Medicine. PR China.

[3]School of Molecular Biosciences, Washington State University, Pullman, WA, 991647520, USA.

[*]Correspondence: liuhuaiwei@sdu.edu.cn.

The authors declare no competing financial interests in relation to this work.

Including Supplemental methods, Table S1—S2, and Figure S1—S17,

SUPPLEMENTAL METHODS

MTO expression and purification

MTO expression plasmids (see Table S1) were constructed using primers listed in Table S2. These plasmids were introduced into *E. coli* BL21 (DE3) and the strains were incubated in LB medium containing kanamycin (50 μg/ml). When OD_{600} reached 0.6, 0.4 mM isopropyl β-D-1-thiogalactopyranoside (IPTG) was added and the temperature was decreased to 25℃. The cultivation was further continued for 16 h. Cells were harvested by centrifugation and then re-suspended in binding buffer (200 mM NaCl, 50 mM NaH_2PO_4, 20 mM Imidazol, pH 8.0).

For protein purification, cell disruption was performed using a Pressure Cell Homogeniser (SPCH-18) at 4℃. Cell lysate was centrifuged to remove the debris and RpMTO was purified using the nickel-nitrilotriacetic acid (Ni-NTA) agarose resin (Invitrogen, Waltham, MA, USA). Purification was conducted by following the manufacturer's instructions. The eluted protein was loaded onto the PD-10 desalting column (GE) for buffer exchange to sodium phosphate buffer (20 mM, pH 7.6). Purity of the proteins was examined via SDS-PAGE.

Quantification of H_2O_2

H_2O_2 generated from MTO catalyzing reaction was measured using both the Hydrogen Peroxide Assay Kit and HRP-catalyzed ADHP method. Mechanism of the former is letting H_2O_2 oxidize Fe^{2+} to Fe^{3+}, which then reacts with xylenol orange to generate purple compounds that have absorbance at 560 nM (A560). The experiment

was performed following the manufacturers instruction.

The HRP-catalyzed ADHP method was performed as below: salt-free lyophilized HRP powder was dissolved in ddH_2O to make a stock solution (4 μg /ml) and HRP working solution was 0.1 μg/ml. ADHP stock solution (15 mM) was prepared by dissolving the ADHP powder in DMSO at room temperature and ADHP working solution was 150 μM. In the reaction system, 200 μL sample was mixed with 5 μL HRP stock solution and 2 μL ADHP stock solution, and then incubated at room temperature for 5 min. The protein was then precipitated down by adding 0.3 mL acetonitrile and was removed by centrifugation (12,000×*g*, 3 min). The supernatant was subjected to fluorescence analysis. The mechanism is ADHP can be catalyzed by HRP to produce resorufin that has fluorescence (λ_{ex}= 571 nm and λ_{em} = 585 nm). The fluorescence was measured with a fluorescent microplate reader. For both methods, commercial H_2O_2 was used to make a standard curve for quantification.

GC-MS quantification of MT

Decane (5.7 ml) was used to extract the remaining MT in reaction system (0.3 ml, the ratio of decane to sample was 19:1, v/v). The extraction was performed at room temperature for 20 min. After extraction, upper layer was pipetted out and subjected to GC-MS analysis using the Q Exactive™ GC Orbitrap™ GC-MS/MS system equipped with a TraceGOLD TG-5SIlMS GC Column (Thermo Fisher Scientific, Waltham, MA, USA). The device was heated from room temperature to 35°C to 60°C to 230°C in 21 minutes. High purity helium (99.999%, V/V) was used as the carrier

gas at a flow rate of 20 mL/min. Full-scan MS spectra (from 32.5 to 500 m/z) were detected.

LC-ESI-MS Analysis of sulfur products

After enzymatic reaction, 50 μL reaction mixture was derivatized with 5 μL mBBr following a previously reported protocol (1). Derivatives were then subjected to LC-ESI-MS analysis. The quadrupole time-of-flight, high-resolution mass spectrometer (Ultimate 3000, Burker impact HD, Thermo Fisher, Waltham, MA, USA) was used. The column was InertSustain C18 5 μm (4.6 mm × 250 mm, GL Sciences, Shanghai, China). Injection volume of sample was 10 μL. The source temperature was set at 200°C and the ion spray voltage was at 4.5 kv. Nitrogen was used as the nebulizer and drying gas. The mobile phase was a mixture of pure water and methanol. A linear gradient of solvent A (0.25% acetic acid in ddH$_2$O) and solvent B (100% methanol) from 7.5% to 100% to 7.5% (solvent B) in 31 min was used for elution, the column temperature was maintained at 38°C. Data were acquired in full MS (100−1500 m/z) mode. Full mass resolution (40000@ m/z 1222) at full sensitivity.

Formaldehyde quantification

Formaldehyde produced in the reaction system was quantified using 4-amino-3-hydrazino-5-mercapto-1,2,4-triazole (Purpald) as previously described (2). The Purpald working solution was 135 mM. Purpald working solution (0.1 ml) was reacted with 0.2 ml sample. The reaction was performed at room temperature for 15 min and then the

absorbance of 550 nm (A_{550}) was measured. Commercial formaldehyde was used to make a standard curve for quantification.

Deletion of mtoX in R. pomeroyi DSS-3

R. pomeroyi DSS-3 Δ*mtoX* was constructed using previously reported methods (3, 4). Briefly, upstream and downstream fragments of *mtoX* were amplified by PCR. These two fragments were ligated with the linearized plasmid pK18mobsacB to construct a deletion plasmid. The deletion plasmid was transformed into *E. coli* MFD pir and then transferred to *R. pomeroyi* DSS-3 by conjugation. The transformed *R. pomeroyi* colonies were confirmed through colony PCR and DNA sequencing. All primers used in the deletion process are listed in Supplemental Table S2.

LC-MS/MS analysis of MTO

MT treated or untreated MTO was digested by trypsin (Promega, Madison, WI, USA) for 12 h at 37°C and passed C18 Zip-Tip (Millipore) for desalting. The Prominence nano-LC system (Shimadzu, Kyoto, Kyoto, Japan) and LTQ-OrbitrapVelos Pro CID mass spectrometer (Thermo Fisher Scientific, Waltham, MA, USA) were used. A linear gradient of solvent A (0.1% formic acid in 2% acetonitrile) and solvent B (0.1% formic acid in 98% acetonitrile) from 0% to 100% (solvent B) in 100 min was used for elution. Full-scan MS spectra (from 400 to 1800 *m*/*z*) were detected with a resolution of 60,000 at 400 *m/z*.

Table S1. Strains and plasmids used in this study

Strain or plasmid	Characteristic or target protein	Source
Strains		
R. pomeroyi DSS-3	wide type, used to amplied the RpMTO fragments	Our lab
R. pomeroyi DSS-3 Δ*pdo*	*pdo* deleted	Our lab
R. pomeroyi DSS-3 Δ*sqr*Δ*fccAB*	*sqr, fccA* and *fccB* deleted	Our lab
R. pomeroyi DSS-3 Δ*mto*	*mto* deleted	This study
MFD *pir*	*E. coli* strain for performing biparental mating to transfer plasmids to other bacteria	Our lab
E. coli XL1-Blue MRF'	Used for plasmid construction	Our lab
BL21(DE3)	Used for protein expression	Our lab
Roseobacter denitrificans OCh114	Used to amplied the RdMTO fragments	Our lab
Ruegeria lacuscaerulensis ITI-1157	Used to amplied the RlMTO fragments	
Plasmids		
pK18mobsacB	Widely used gene-knockout vector, kanamycin resistance	This study
pK18mobsacB$_{tet}$-Δ*mto*	pK18mobsacB containing the upstream and downstream sequence of the *mto* gene	This study
pET28a-RpMTO	Used to express the RpMTO protein	This study
pET28a-RdMTO	Used to express the RdMTO protein	This study
pET28a-RlMTO	Used to express the RlMTO protein	This study
pET28a-RpMTO C28S	The Cys^{28} of RpMTO was mutated to Ser	This study
pET28a-RdMTO C82S	The Cys^{82} of RdMTO was mutated to Ser	This study
pET28a-RdMTO C143S	The Cys^{143} of RdMTO was mutated to Ser	This study
pET28a-RdMTO C448S	The Cys^{448} of RdMTO was mutated to Ser	This study
pET28a-RdMTO C82SC143S	The Cys^{82} and Cys^{143} of RpMTO were mutated to Ser	This study
pET28a-RdMTO C143SC448S	The Cys^{143} and Cys^{448} of RpMTO were mutated to Ser	This study
pET28a-RdMTO C82SC448S	The Cys^{82} and Cys^{448} of RpMTO were mutated to Ser	This study
pET28a-RdMTO C82SC143SC448S	The Cys^{82}, Cys^{143} and Cys^{448} of RpMTO were mutated to Ser	This study
pET28a-RlMTO C76S	The Cys^{76} of RlMTO was mutated to Ser	This study
pET28a-RlMTO C140S	The Cys^{140} of RlMTO was mutated to Ser	This study
pET28a-RlMTO C456S	The Cys^{456} of RlMTO was mutated to Ser	This study

Table S2. Primers used in this study

Primer name	Sequence (5'-3')	Descriptions
mto-Up-F	AATTCCTGGCCGTGTTCATCGCAGC	
mto-Up-R	GAGACTTTCCTCCCGTTTGGATCGTTAATGCGT	
mto-Down-F	CAAACGGGAGGAAAGTCTCCCTCCCTGACTTCCG	
mto-Down-R	TACCGAGCTCGAATTGCGCCCCAGTTCGATCTT	
mto-V-F	GATCTGATCCATATCGAGACACCG	*mto* deletion
mto-V-R	CAAAGCTGATGGTCAACAGCTG	
pK18mobsacB-F	GCGCAATTCGAGCTCGGTACCCGG	
pK18mobsacB-R	GATGAACACGGCCAGGAATTCGTAATCATGTCATAG	
PET28a-RpMTO-F	TTCGTCCTGAAAGCTTGCGGCCGCACTC	
PET28a-RpMTO-R	CTTTTCATATGGCTGCCGCGCGGCAC	RpMTO
RpMTO-F	CGCGGCAGCCATATGAAAAGAAGAGAATTTG	expression
RpMTO-R	CCGCAAGCTTTCAGGACGAATAGAGCGC	
PET28a-RdMTO-F	CTGGATTTGAAAGCTTGCGGCCGCACTC	
PET28a-RdMTO-R	TCTGTCATATGGCTGCCGCGCGGCAC	RdMTO
RdMTO-F	GCGGCAGCCATATGACAGAGACAAACGGAAGC	expression
RdMTO-R	CGCAAGCTTTCAAATCCAGATGTCGGATGAGCAATC	
PET28a-RlMTO-F	GAGCGTGTGAAAGCTTGCGGCCGCACTC	
PET28a-RlMTO-R	TTGTTCATATGGCTGCCGCGCGGCAC	RlMTO
RlMTO-F	GCGGCAGCCATATGAACAAGCGCCCGGA	expression
RlMTO-R	CGCAAGCTTTCACACGCTCGGATAACAAAAAGAGT	
YZ-pET-F	GATCCCGCGAAATTAATACGACTC	Primers for
YZ-pET-R	GGCGCGTCCCATTCGCCA	sequencing
RpMTO-C28S-1-F	GACGAAACCAGTCAATCGCCCTATATG	
RpMTO-C28S-2-R	GCGATTGACTGGTTTCGTCGGCAAAG	RpMTO C28S
RpMTO-C28S-2-F	GAGATACCTACAGCGTGAGC	expression
RpMTO-C28S-1-R	GCTCACGCTGTAGGTATCTC	

C82S-1-F	GAACGCCAGTTCTTCGTGCAACACC	
C82S-2-R	GCAGGAAGAACTGGCGTTCCAACCCATG	
RdMTO-2-F	GTTCTTTCCTGCGTTATCC	
RdMTO-1-R	GGATAACGCAGGAAAGAAC	Cysteine-mutated RdMTO expression
C143S-1-F	GGTGCATTCTCTTGGTGCCGATATC	
C143S-2-R	GGCACCAAGAGAATGCACCGTGTGGGG	
C448S-1-F	GGCGGTGATTCCTCATCCGACATCTG	
C448S-2-R	GGATGAGGAATCACCGCCGGGATAGC	
RlMTO-2-F	GCTTGGAGCGAACGACCTACACCG	
RlMTO-1-R	CGGTGTAGGTCGTTCGCTCCAAGC	
C76-1-F	GAACGCCTCCAGCAGCGCCTTGAG	
C76-2-R	CTCAAGGCGCTGCTGGAGGCGTTC	Cysteine-mutated RlMTO expression
C140-1-F	AATTCATTCCGGCCCGGAGGGCAT	
C140-1-R	ATGCCCTCCGGGCCGGAATGAATT	
C456-1-F	GCGGCGATTCTAGCACCGACTCTT	
C456-2-R	AAGAGTCGGTGCTAGAATCGCCGC	

Underlining represents the sequences of homologous arms.

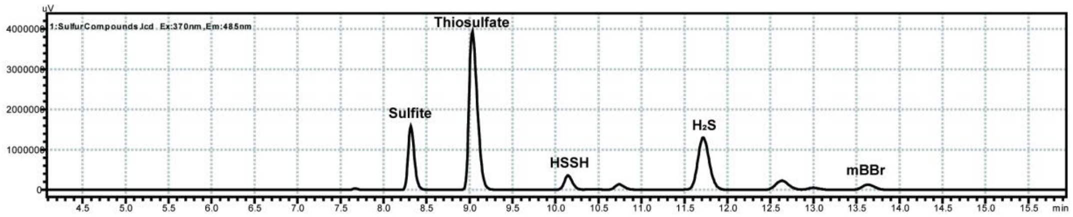

Figure S1. HPLC analysis of the products from chemical reaction of H_2S with H_2O_2.

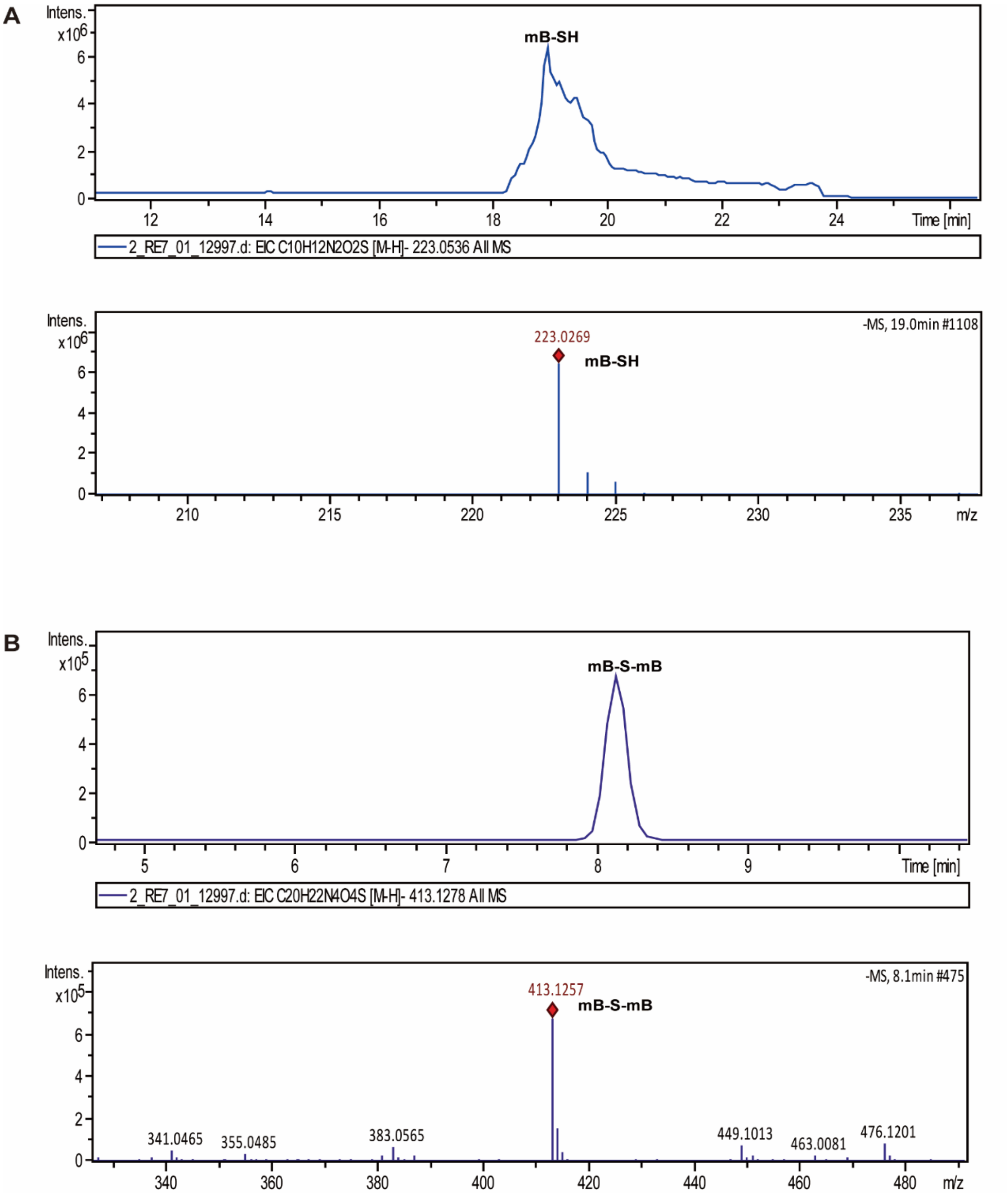

Figure S2. MS spectra of the H_2S derivatives from RpMTO catalyzed methanethiol degradation.

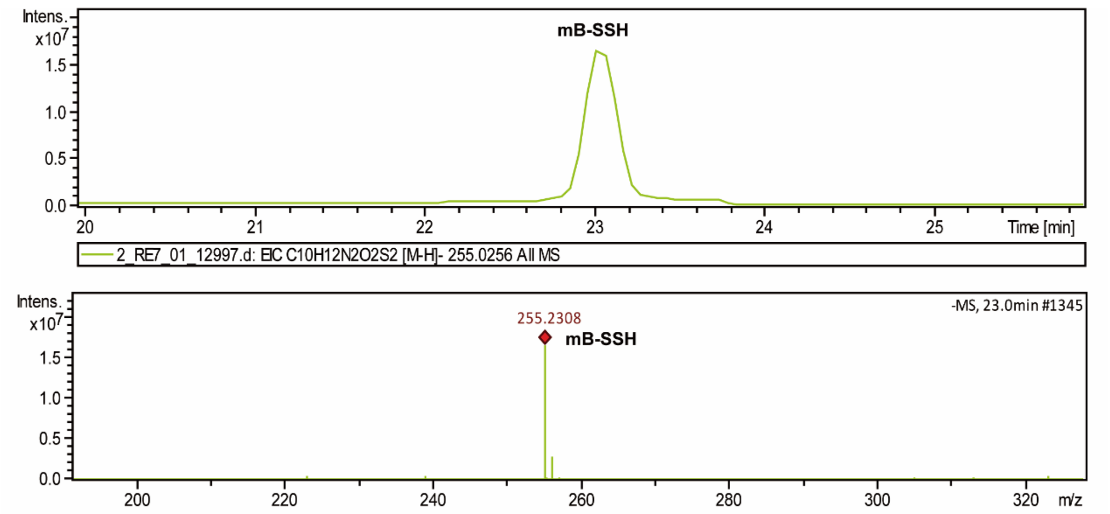

Figure S3. MS spectra of the HSSH derivative from RpMTO catalyzed methanethiol degradation.

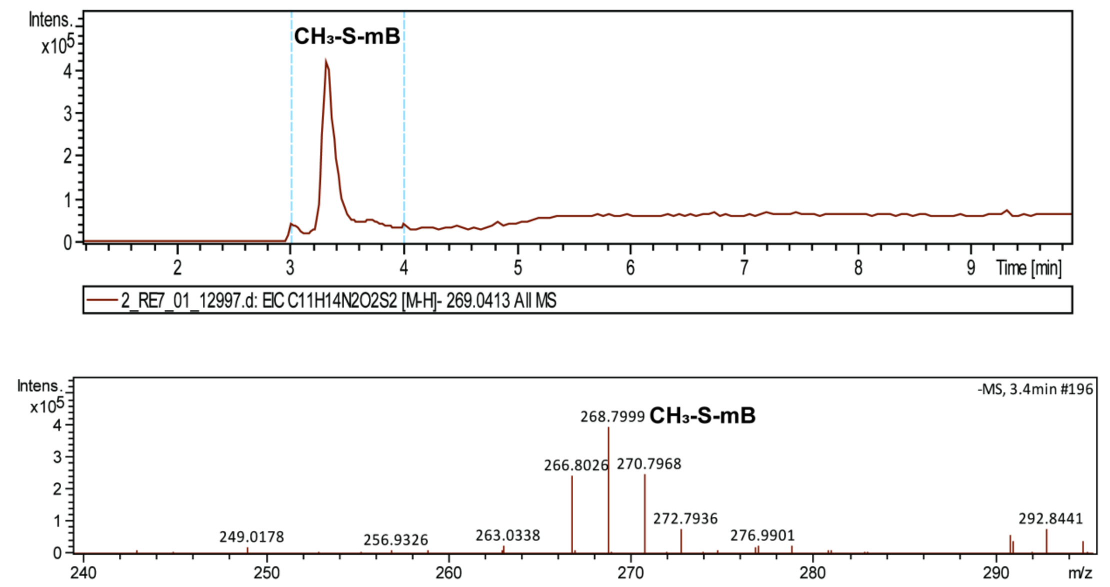

Figure S4. MS spectra of the CH_3SH derivative from RpMTO catalyzed methanethiol degradation.

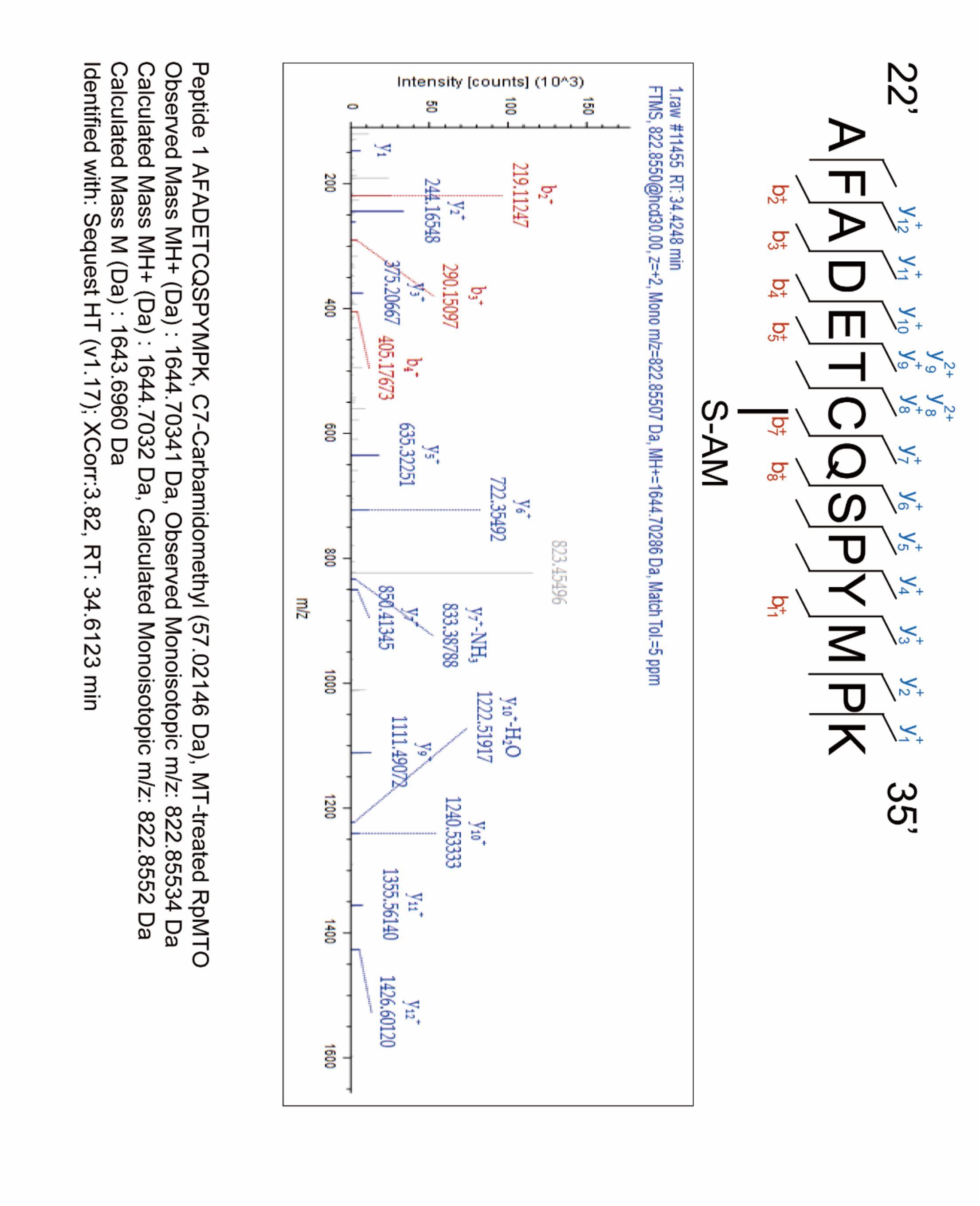

Figure S5. MS^2 spectra of peptide 1 (Cys28-S-AM) (from MT-treated RpMTO).

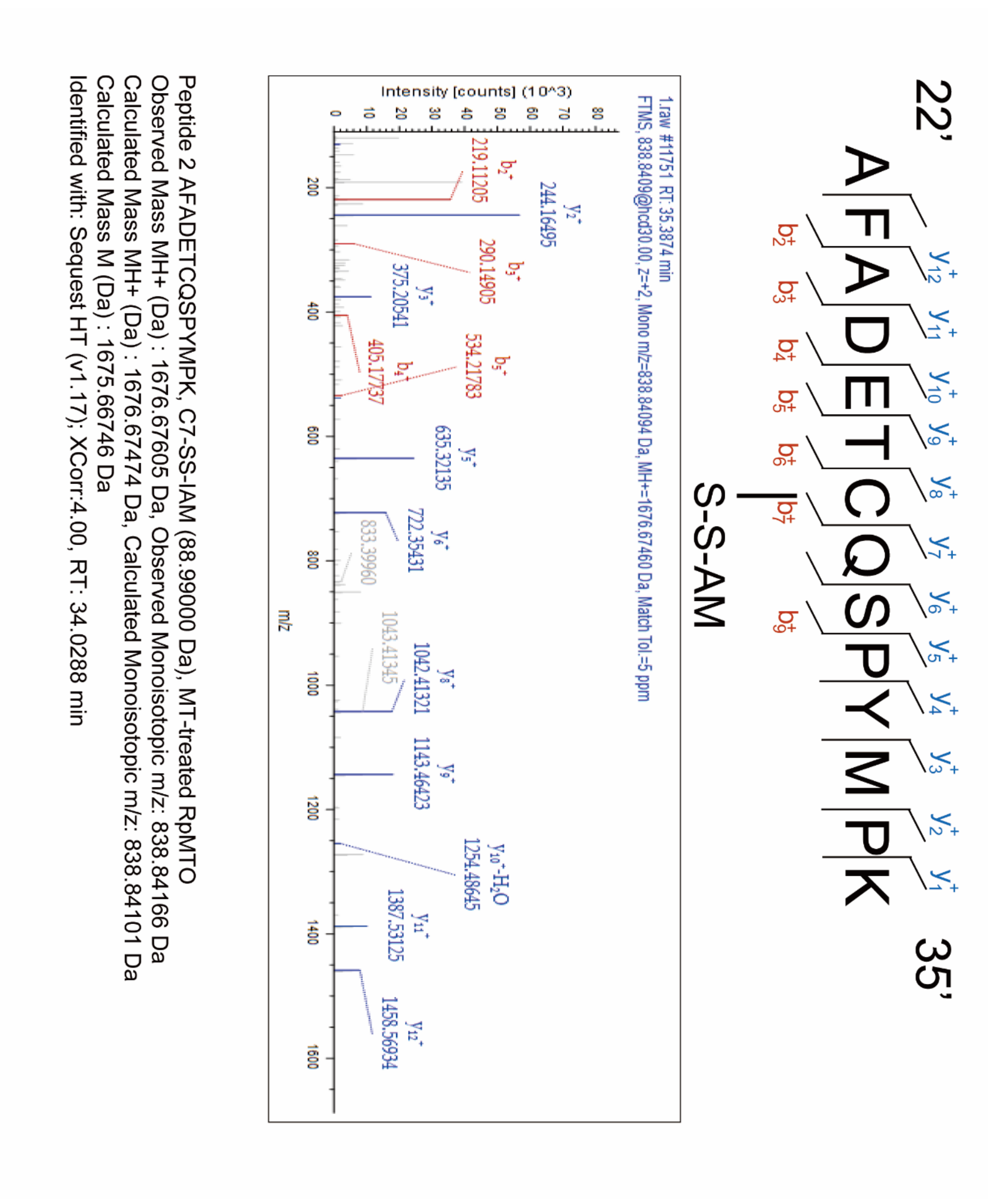

Figure S6. MS^2 spectra of peptide 2 (Cys28-S-S-AM) (from MT-treated RpMTO).

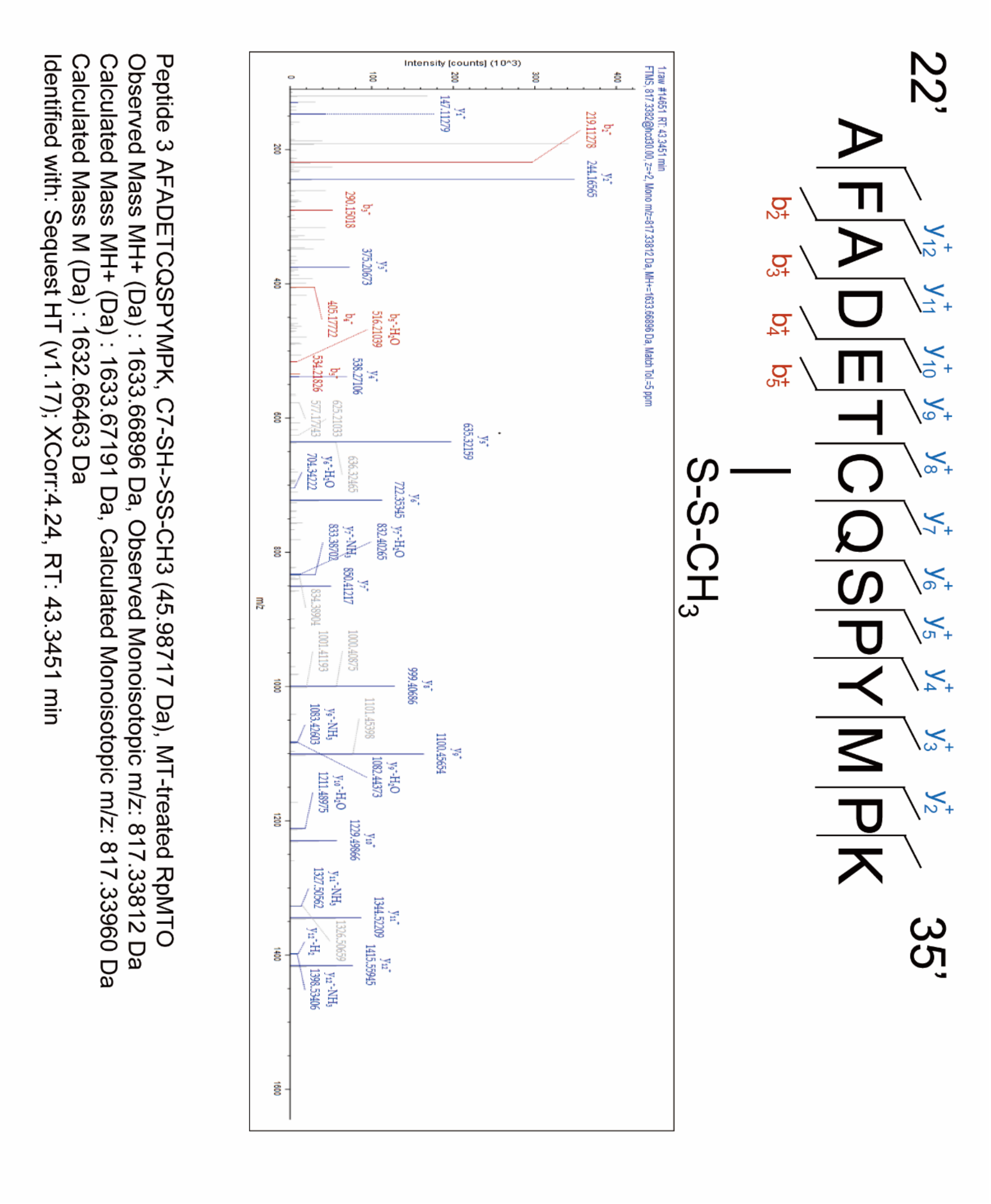

Figure S7. MS^2 spectra of peptide 3 (Cys28-S-S-CH_3) (from MT-treated RpMTO).

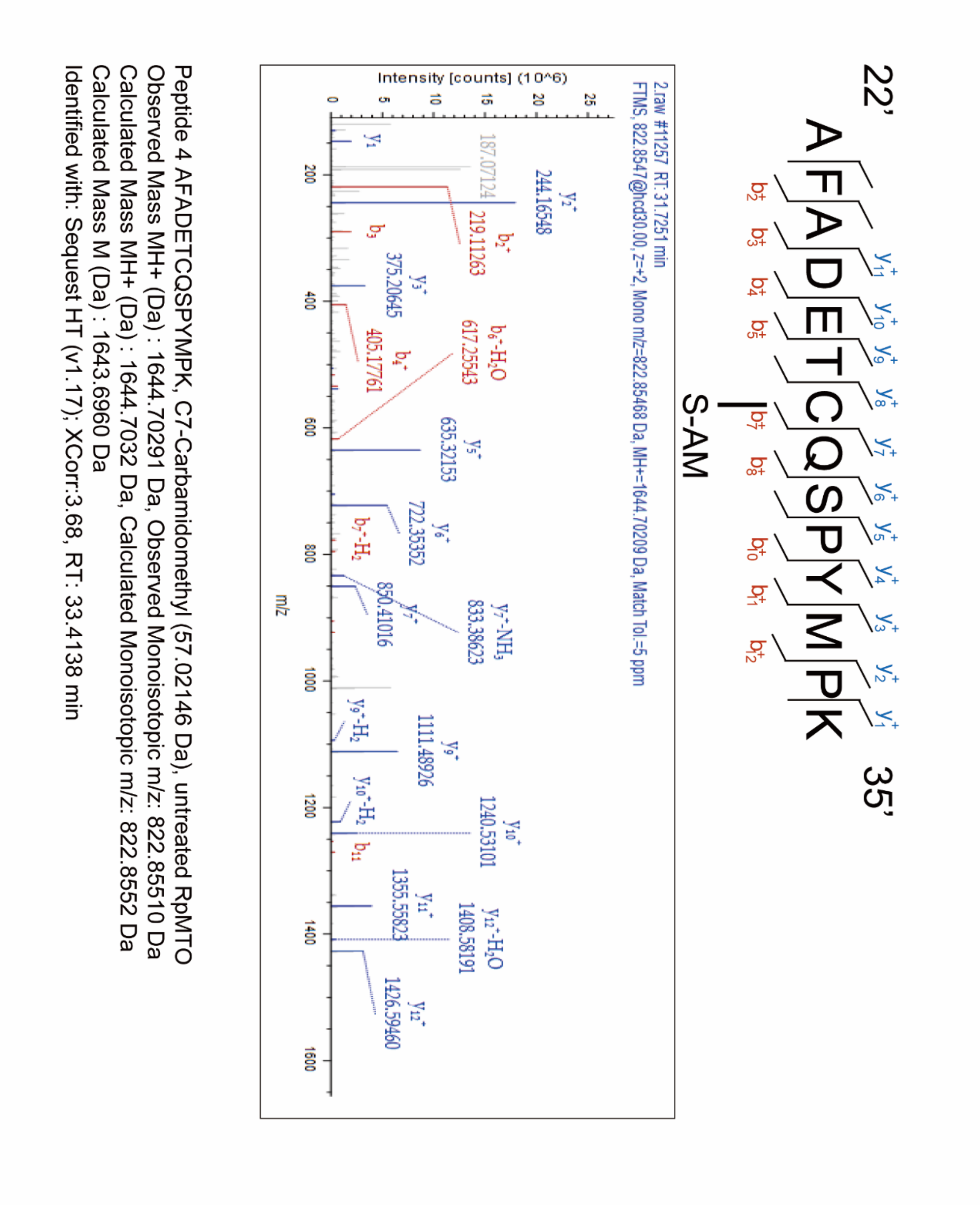

Figure S8. MS^2 spectra of peptide 4 (Cys28-S-AM) (from untreated RpMTO).

```
                    1        10        20        30        40        50
WP_011242048.1  .MKRREFG.ALAAGALAMGLPFRAFADETCQSPYMPKITGQEEFVYVWTLGVEGMGDEQD
WP_072505285.1  .MNRREFGGLTAATALAMAGPFRAFADETCQSPYMPKITGQEEFVYVWTLGVEGMGDEQD
WP_106474101.1  .MNRREFSTLAASAMVLGGSPFRAFADETCQSPYMPKITGTEEFIYVWTLGVEGMGDEQD
WP_039527425.1  .MNRREFG.LLSATWLAAGLPTGLRADETCQSPYMPKITGHEEFVYVWTLGVEGMGDGQD
WP_027263838.1  .MKRREFGGMAVSALALTAMPFSVSADETCQSPYMPMITGHEEYVYIWTLGVEGMGDEQD
WP_138163781.1  .MKRRQFGALATAALVMGGP.LRALADETCQSPYMPKITGHEEYVYIWTLGVEGMGDGQD
WP_218448412.1  .........MAATSAIAVASAFGAARADETCQSPYMPKITGHEEFVYVWTLGVEGMGDEQD
WP_036733147.1  MRRRDFNLFAAAAAGGFVLRPGLGFADGTCQSPYMVRIEGREQFVYVWTLGKEGVGDGQD
                                              *

                60        70        80        90        100       110
WP_011242048.1  KLVTIDLRPGSATRGQVINSVSVGGRNEAHHGGFSADRRFFWTGGLDTNRIFIFDVHSDP
WP_072505285.1  KMVTVDLRPGSPTRGEVIHSLSVGGRNEAHHGGFSADRRYFWTGGLDTNRIFIFDIHSDP
WP_106474101.1  KLVTIDARPDSSTRGQVINSVSVGGRNEAHHGGFNSDRRYFWTGGLDTNRIFIFDVHSDP
WP_039527425.1  KLVTVDVRPNSPTKGTVINSVSVGGRNEAHHGGFSADRRYFWAGGLDTNRIFIFDVHSDP
WP_027263838.1  KMVTVDLRPNSDTRGQVINSVSVGGRNEAHHAGFNSDRRYLWAGGLDTNRIFIFDVHSDP
WP_138163781.1  KMVTVDLRPGSETRGQVINSLSIGGRNEAHHAGFSADRRYLWAGGLDTNRIFIFDVHSDP
WP_218448412.1  KLVTIDLRPGSPTRGQVIASLSVGGRNEAHHGGFSSDRRYYWAGGLDTNRIFIFDVHSDP
WP_036733147.1  KLVTVDLRDGSPTRGKIVDSLSVGGRNEAHHGGFTADRRHYWTGGLDTNRIFIFDVASDA

              120       130       140       150       160       170
WP_011242048.1  SNPKLHKTIDTFVKDSGGVVGPHTFFALPGSMMITGLSNDDDHGGRTALVEYNDD.GEYV
WP_072505285.1  AAPKLHKVIETFVTDSGGVVGPHTFFALPGSMMITGLSNQDDHGGRTALVEYNDD.GDYV
WP_106474101.1  STPKLHKTIDTFVKDSGGVVGPHTFFALPGRMMISGLSNDGDHGGRTALVEYSDE.GDYL
WP_039527425.1  SQPRLHKTIETFVEDSGGVVGPHTFFALPGRMLITGLSNSDDHGGRTALVEYNDD.GDYL
WP_027263838.1  SKPSLHKTIDSFVKDSGGCVGPHTFFALPGRMMITGLSNDEDHGGRTALVEYNDD.GEYV
WP_138163781.1  ANPVLHKTIDTFVKDADGPVGPHTFFALPGRMMITALSNDDDHGGRTALVEYTDE.GDFV
WP_218448412.1  AKPTLHKTIETFVSDSGGFVGPHTFFAVPGRMLITALSNDQDHGGRTALVEYNDD.GEYI
WP_036733147.1  GKPKLVKTIDSFVKDSGGVVGPPTFFALPGKMLTSGLSNDQDHGGRTAIVEYTDDGAQCL

                180       190       200       210       220       230
WP_011242048.1  ATYWMPTADDMQGAVAVGDAVADGYGYDIRALIRKNVMLTSSFTGWSNYMMDFGQMLQDA
WP_072505285.1  ATYWMPTADDMQGAVAVDGAVADGYGYDIRALIRKNIMLTSSFTGWSNYMMDFGQMLQDA
WP_106474101.1  ATYWHPTAEDMQGAEAVGDAVADGFGYDIRALIRKNIMLTSSFTGWSNYMMDFGQMLQDG
WP_039527425.1  ATYWMPTADDMRGAVAVDGAVADGYGYDIRALIRKNIMLTSSFTGWSNYMMDFGQMLQDP
WP_027263838.1  ATYWMPTADDMQGAVAADGAVADGYGYDVRALIRKNIMLTSSFTGWSNYMMDFGQMLGDA
WP_138163781.1  ETHWMPTAEDMRGAEAVDGAVADGFGYDVRALIRKNVMLTSSFTGWSNYMMDFGQMLGDS
WP_218448412.1  ATYPIPTESDLQGAVKVDGAVADGFGYDVRALIRKNVMLTSSFTGWSNYMMDFGQMLQDA
WP_036733147.1  GTYWMPTADDMRGAVAVDGALADGYGYDLRARPDRNVMLTSSFTGWSNYMMDFGQMLQAP

                240       250       260       270       280       290
WP_011242048.1  EAMKRFGNTIVQWDLHTRQPKKVFNVPGAPLEIRFPWGSNANYAFSTTALTSQLWLIYED
WP_072505285.1  EAMKRFGNSMVLWDLHTRQPRKVFNVPGAPLEVRFPWGPNANYAFSTTALTSQLWLIYED
WP_106474101.1  EAMKRFGNTVVQWDLHTRQPKKVFNVPGAPLEIRFPWGANANYAFTSAALTSKLWLIYED
WP_039527425.1  EAMQRFGNTIVQWDLHTRQPRKVFNVPGAPLEIRFPWGSNANYAFSSTALTSQLWLIYED
WP_027263838.1  EAMKRFGGTMVQWDLHTRQPKKVFSVPGAPLEVRFPWGPNANYAFSTTALTSKLWLIYED
WP_138163781.1  EAMKRFGSTIVQWDLHTRQPKKVFNVPGAPLEIRFPWGPNANYAFSTTALTSQLWLIHED
WP_218448412.1  EAMKRFGNTIVQWDLHTRQPKKVFNVPGAPLEIRFAWGANANYAFSTTALTSKLWLIYED
WP_036733147.1  EAMKRFGNTVVQWDLHARQPRRVFNVPGAPLELRFAWGEGHNYAFTTSALTSAMWLIFED

                300       310       320       330       340       350
WP_011242048.1  DAGEWQAKAVADIGNPADIPLPVDISIAADDQTLWINSFMDGKTRLFDISDPHKPFQIYE
WP_072505285.1  DHGEWQAKSVADIGNPEDIPLPVDISIAADDQTLWVNSFMDGKTRLFDISDPHHPSQIYE
WP_106474101.1  DGGEWQAKEVADIGNPADIPLPVDISIAADDQTLWVNSFMDGKTRLFDISDPHNPSQIYE
WP_039527425.1  DAGEWQAKAVADIGNPADIPLPVDISIAADDQTLWVNSFMDGKTRLFDVSDPHNPKQVYE
WP_027263838.1  DAGEWQAKAVADIGNPADIPLPVDISLAADDQTLWINSFMDGTTRLFDVSDPHNPKQIYE
WP_138163781.1  DTGEWQAKAVADIGDPTKIPLPVDISLAADDQTLWINTFLDGMTRLFDVSDPHHPKQIYE
WP_218448412.1  EDGEWQAKDVGDIGNPADIPLPVDISITADDQHLWVNTFMDGKTRLFDVSDPHNPKQIYE
WP_036733147.1  DAGVWQAEKVADVGDPSKIPLPVDISIAADDQTLWVNTFMDGTTRLFDISDPHKPMMVYE

                360       370       380       390       400       410
WP_011242048.1  KVIDRQVNMVSQSWDGKRVYFSSSLLANWDKKGKDDAQYLKAYNWDGKELVEDFAVDFYE
WP_072505285.1  KTIDRQVNMVSQSWDGKRVYFSSSLLANWDKKGDDDVQYLRAYNWDGKELVEDFNIDFYA
WP_106474101.1  KQIGRQVNMVSQSWDGSRVYYTSSLLSQWDKKGEDDEQYLKAYSWDGTELTEDYSVDFYE
WP_039527425.1  KVIGPQVNMVSQSWDGQRVYFTSSLLANWDKKGEDNAQYLKAYSWDGNELVEDFEIDFNA
WP_027263838.1  KKIASQVNMVSQSWDGERVYYTSSLLGNWDKKGADDAQYLKAYKWNGSELVDDFEIDFYE
WP_138163781.1  KKIASQVNMVSQSWDGNRIYFTSSLLANWDKKGADDQQYLKAFTWNGSELVPDFELDFYE
WP_218448412.1  KVIGAQVNMVSQSWDGKRVYFTSSLLANWDKKGAADEQYLKAYTWDGSELVEDFSIDFYK
WP_036733147.1  KVIGPQVNMVSQSWDGKRVYFTTSLLGNWDKKAGDDVQFLKAYTWDGKELVEDFAIDFYA
```

```
               420       430
WP_011242048.1 LGLGRAHIMRFGSSALYSS
WP_072505285.1 AGLGRAHIMRFGSSALYSA
WP_106474101.1 LKLGRAHIMRFGSSALYTS
WP_039527425.1 LNLGRAHIMRFGSAALYA.
WP_027263838.1 AQLGRAHLMRFGSAALYS.
WP_138163781.1 LQLGRAHLMRFGSSELYSA
WP_218448412.1 LNLGRAHIMRFGSSKLYTS
WP_036733147.1 EGMGRAHMMNFGSAELFSA
```

Figure S9. Amino acid sequence alignment of 8 cluster 2 MTOs. Conserved cysteine residue is marked with asterisk (*).

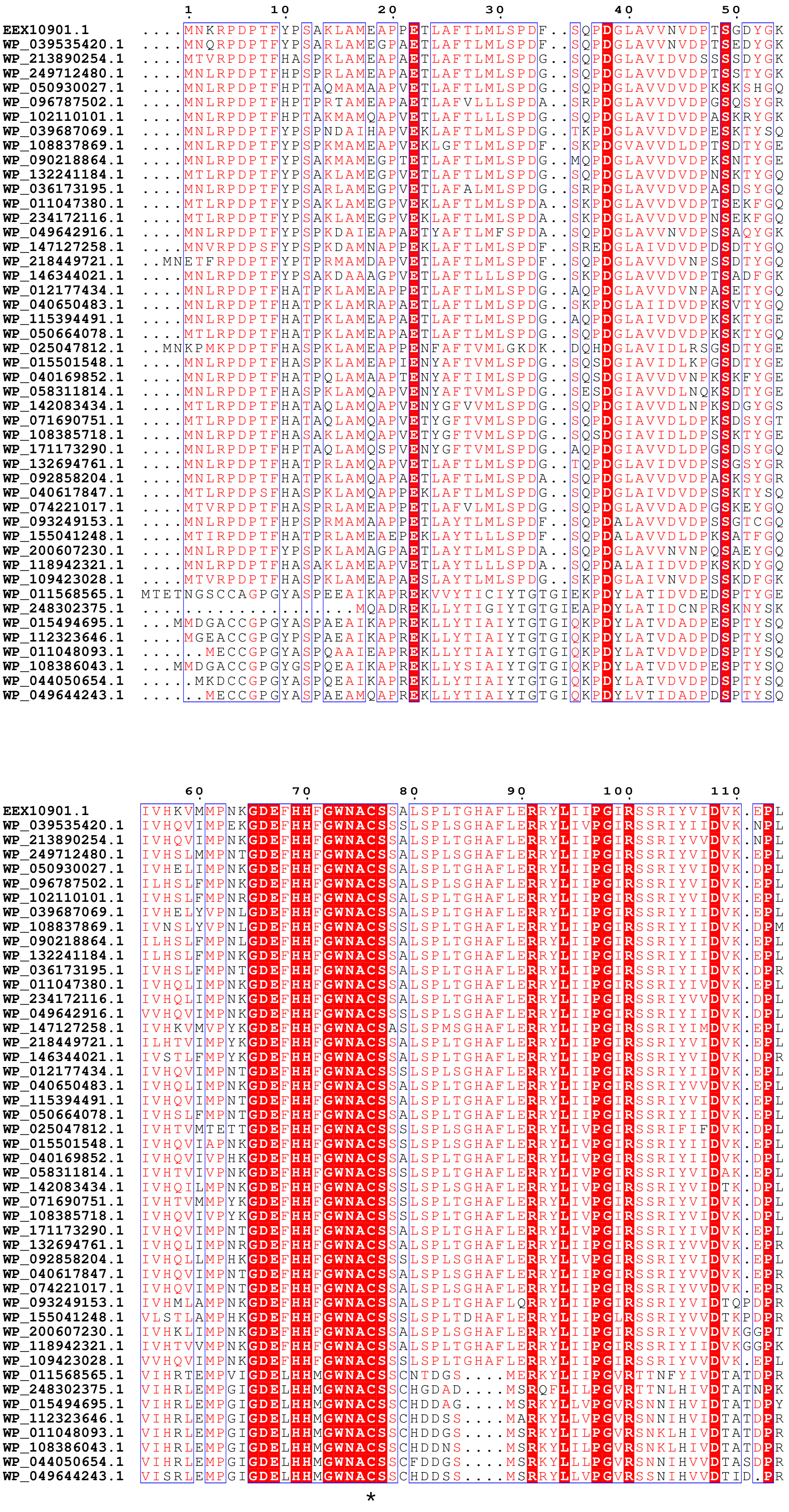
1 10 20 30 40 50
EEX10901.1 ....MNKRPDPTFYPSAKLAMEAPPETLAFTLMLSPDF..SQPDGLAVVNVDPTSGDYGK
WP_039535420.1 ....MNQRPDPTFYPSARLAMEGPAETLAFTLMLSPDF..SQPDGLAVVNVDPTSEDYGK
WP_213890254.1 ....MTVRPDPTFHASPKLAMEAPAETLAFTLMLSPDG..SQPDGLAVIDVDSSSSDYGK
WP_249712480.1 ....MNLRPDPTFHPSPRLAMEAPAETLAFTLMLSPDG..SQPDGLAVVDVDPTSSTYGK
WP_050930027.1 ....MNLRPDPTFHPTAQMAMAAPVETLAFTLMLSPDG..SQPDGLAVVDVDPKSKSHGQ
WP_096787502.1 ....MNLRPDPTFHPTPRTAMEAPAETLAFVLLLSPDA..SRPDGLAVVDVDPGSQSYGR
WP_102110101.1 ....MNLRPDPTFHPTAKMAMQAPVETLAFTLLLSPDG..SQPDGLAVIDVDPASKRYGK
WP_039687069.1 ....MNLRPDPTFYPSPNDAIHAPVEKLAFTLMLSPDG..TKPDGLAVVDVDPESKTYSQ
WP_108837869.1 ....MNLRPDPTFYPSPKMAMEAPVEKLGFTLMLSPDF..SKPDGVAVVDLDPTSDTYGE
WP_090218864.1 ....MNLRPDPTFYPSAKMAMEGPTETLAFTLMLSPDG..MQPDGLAVVDVDPKSNTYGE
WP_132241184.1 ....MTLRPDPTFYPSAKLAMEGPVETLAFTLMLSPDG..SQPDGLAVVDVDPNSKTYGQ
WP_036173195.1 ....MNLRPDPTFYPSARLAMEGPVETLAFALMLSPDG..SRPDGLAVVDVDPASDSYGQ
WP_011047380.1 ....MTLRPDPTFYPSAKLAMEGPVEKLAFTLMLSPDA..SKPDGLAVVDVDPTSEKFGQ
WP_234172116.1 ....MTLRPDPTFYPSAKLAMEGPVEKLAFTLMLSPDA..SKPDGLAVVDVDPNSEKFGQ
WP_049642916.1 ....MNLRPDPTFYPSPKDAIEAPAETYAFTLMFSPDA..SQPDGLAVVNVDPSSAQYGK
WP_147127258.1 ....MNVRPDPSFYPSPKDAMNAPPEKLAFTLMLSPDF..SREDGLAIVDVDPDSDTYGQ
WP_218449721.1 ..MNETFRPDPTFYPTPRMAMDAPVETLAFTLMLSPDF..SQPDGLAVVDVNPSSDTYGQ
WP_146344021.1 ....MNLRPDPTFYPSAKDAAGPVETLAFTLLLSPDG..SKPDGLAVVDVDPTSADFGK
WP_012177434.1 ....MNLRPDPTFHATPKLAMEAPPETLAFTLMLSPDG..AQPDGLAVVDVNPASETYGQ
WP_040650483.1 ....MNLRPDPTFHATPKLAMRAPAETLAFTLMLSPDG..SKPDGLAIIDVDPKSVTYGQ
WP_115394491.1 ....MNLRPDPTFHATPKLAMEAPAETLAFTLMLSPDG..AQPDGLAIVDVDPKSKTYGE
WP_050664078.1 ....MTLRPDPTFHATPKLAMEAPAETLAFTLMLSPDG..SQPDGLAVVDVDPKSKTYGQ
WP_025047812.1 ..MNKPMKPDPTFHASPKLAMEAPPENFAFTVMLGKDK..DQHDGLAVIDLRSGSDTYGE
WP_015501548.1 ....MNLRPDPTFHASPKLAMEAPIENYAFTVMLSPDG..SQSDGIAVIDLKPGSDTYGE
WP_040169852.1 ....MNLRPDPTFHATPQLAMAAPTENYAFTIMLSPDG..SQSDGIAVVDVNPKSKFYGE
WP_058311814.1 ....MNLRPDPTFHASPKLAMQAPVENYAFTVMLSPDG..SESDGIAVVDLNQKSDTYGE
WP_142083434.1 ....MTLRPDPTFHATAQLAMQAPVENYGFVVMLSPDG..SQPDGIAVVDLNPKSDGYGS
WP_071690751.1 ....MTLRPDPTFHATAQLAMQAPVETYGFTVMLSPDG..SQPDGIAVVDLDPKSDSYGT
WP_108385718.1 ....MNLRPDPTFHASAKLAMQAPVETYGFTVMLSPDG..SQSDGIAVIDLDPSSKTYGE
WP_171173290.1 ....MNLRPDPTFHPTAQLAMQSPVENYGFTVMLSPDG..AQPDGIAVVDLDPGSDSYGQ
WP_132694761.1 ....MNLRPDPTFHATPRLAMQAPVETLAFTLMLSPDG..TQPDGLAVIDVDPSSGSYGR
WP_092858204.1 ....MNLRPDPTFHATPKLAMQAPAETLAFTLMLSPDA..SQPDGLAVVDVDPASKSYGR
WP_040617847.1 ....MTLRPDPSFHASPKLAMQAPPEKLAFTLMLSPDG..SQPDGLAIVDVDPSSKTYSQ
WP_074221017.1 ....MTVRPDPTFHASPRLAMEAPPETLAFVLMLSPDG..SQPDGLAVVDADPGSKEYGQ
WP_093249153.1 ....MNLRPDPTFHPSPRMAMAAPPETLAYTLMLSPDF..SQPDALAVVDVDPSSGTCGQ
WP_155041248.1 ....MTIRPDPTFHATPRLAMEAEPEKLAYTLLLSPDF..SQPDALAVVDLDPKSATFGQ
WP_200607230.1 ....MNLRPDPTFYPSPKLAMAGPAETLAYTLMLSPDA..SQPDGLAVVNVNPQSAEYGQ
WP_118942321.1 ....MNLRPDPTFHASAKLAMEAPVETLAYTLLLSPDA..SQPDALAIIDVDPKSKDYGQ
WP_109423028.1 ....MTVRPDPTFHASPKLAMEAPAESLAYTLMLSPDG..SKPDGLAIVNVDPSSKDFGK
WP_011568565.1 MTETNGSCCAGPGYASPEEAIKAPREKVVYTICIYTGTGIEKPDYLATIDVDEDSPTYGE
WP_248302375.1 ....................MQADREKLLYTIGIYTGTGIEAPDYLATIDCNPRSKNYSK
WP_015494695.1 ...MMDGACCGPGYASPAEAIKAPREKLLYTIAIYTGTGIQKPDYLATVDADPESPTYSQ
WP_112323646.1 ....MGEACCGPGYPSPAEAIKAPREKLLYTIAIYTGTGIQKPDYLATVDADPDSPTYSQ
WP_011048093.1 ......MECCGPGYASPQAAIEAPREKLLYTIAIYTGTGIQKPDYLATVDVDPDSPTYSQ
WP_108386043.1 ...MMDGACCGPGYGSPQEAIKAPREKLLYSIAIYTGTGIQKPDYLATVDVDPESPTYSQ
WP_044050654.1 .....MKDCCGPGYASPQEAIKAPREKLLYTIAIYTGTGIQKPDYLATVDVDPDSPTYSQ
WP_049644243.1 ......MECCGPGYASPAEAMQAPREKLLYTIAIYTGTGIQKPDYLVTIDADPDSPTYSQ
60 70 80 90 100 110
EEX10901.1 IVHKVMMPNKGDEFHHFGWNACSSALSPLTGHAFLERRYLIIPGIRSSRIYVIDVK.EPL
WP_039535420.1 IVHQVIMPEKGDEFHHFGWNACSSSLSPLSGHAFLERRYLIVPGIRSSRIYIIDVK.NPL
WP_213890254.1 IVHQVIMPNKGDEFHHFGWNACSSALSPLTGHAFLERRYLIIPGIRSSRIYVVDVK.NPL
WP_249712480.1 IVHSLMMPNTGDEFHHFGWNACSSALSPLSGHAFLERRYLIIPGIRSSRIYVIDVK.EPL
WP_050930027.1 IVHELIMPNKGDEFHHFGWNACSSALSPLTGHAFLERRYLIIPGIRSSRIYVIDVK.DPL
WP_096787502.1 ILHSLFMPNKGDEFHHFGWNACSSALSPLSGHAFLERRYLIIPGIRSSRIYVIDVK.EPL
WP_102110101.1 IVHSLFMPNRGDEFHHFGWNACSSALSPLTGHAFLERRYLIIPGIRSSRIYVIDVK.EPL
WP_039687069.1 IVHELYVPNLGDEFHHFGWNACSSALSPLTGHAFLERRYLIIPGIRSSRIYIIDVK.EPL
WP_108837869.1 IVNSLYVPNLGDEFHHFGWNACSSSLSPLTGHAFLERRYLIIPGIRSSRIYIIDVK.DPM
WP_090218864.1 ILHSLFMPNKGDEFHHFGWNACSSALSPLTGHAFLERRYLIIPGIRSSRIYIIDVK.EPL
WP_132241184.1 ILHSLFMPNKGDEFHHFGWNACSSALSPLTGHAFLERRYLIIPGIRSSRIYIIDVK.EPL
WP_036173195.1 IVHSLFMPNTGDEFHHFGWNACSSALSPLTGHAFLERRYLIIPGIRSSRIYIIDVK.DPR
WP_011047380.1 IVHQLIMPNKGDEFHHFGWNACSSALSPLTGHAFLERRYLIIPGIRSSRIYVIDVK.EPL
WP_234172116.1 IVHQLIMPNKGDEFHHFGWNACSSALSPLTGHAFLERRYLIIPGIRSSRIYVVDVK.DPL
WP_049642916.1 VVHQVIMPNKGDEFHHFGWNACSSSLSPLSGHAFLERRYLIIPGIRSSRIYIIDVK.DPL
WP_147127258.1 IVHKVMVPYKGDEFHHFGWNACSASLSPMSGHAFLERRYLIIPGIRSSRIYIMDVK.EPL
WP_218449721.1 ILHTVIMPYKGDEFHHFGWNACSSALSPLSGHAFLERRYLIIPGIRSSRIYVIDVK.EPL
WP_146344021.1 IVSTLFMPYKGDEFHHFGWNACSSALSPLTGHAFLERRYLIIPGIRSSRIYVIDVK.DPR
WP_012177434.1 IVHQVIMPNTGDEFHHFGWNACSSSLSPLSGHAFLERRYLIIPGIRSSRIYIIDVK.EPL
WP_040650483.1 IVHQLIMPNKGDEFHHFGWNACSSALSPLTGHAFLERRYLIIPGIRSSRIYVVDVK.EPL
WP_115394491.1 IVHQVIMPNTGDEFHHFGWNACSSALSPLTGHAFLERRYLIIPGIRSSRIYVIDVK.EPL
WP_050664078.1 IVHSLFMPNTGDEFHHFGWNACSSALSPLTGHAFLERRYLIIPGIRSSRIYIIDVK.EPL
WP_025047812.1 IVHTVMTETTGDEFHHFGWNACSSSLSPLSGHAFLERRYLIVPGIRSSRIFIFDVK.DPL
WP_015501548.1 IVHQVIAPNKGDEFHHFGWNACSSSLSPLSGHAFLERRYLIVPGIRSSRIYIIDVK.EPL
WP_040169852.1 IVHQVIVPHKGDEFHHFGWNACSSALSPLTGHAFLERRYLIVPGIRSSRIYIIDVK.DPL
WP_058311814.1 IVHTVIVPNKGDEFHHFGWNACSSSLSPLTGHAFLERRYLIVPGIRSSRIYVIDAK.EPL
WP_142083434.1 IVHQILMPNKGDEFHHFGWNACSSSLSPLSGHAFLERRYLIVPGIRSSRIYVIDTK.DPL
WP_071690751.1 IVHTVMMPYKGDEFHHFGWNACSSSLSPLTGHAFLERRYLIVPGIRSSRIYVIDVK.DPL
WP_108385718.1 IVHQVIVPYKGDEFHHFGWNACSSSLSPLTGHAFLERRYLIVPGIRSSRIYIIDVK.DPL
WP_171173290.1 IVHQVIMPNTGDEFHHFGWNACSSALSPLTGHAFLERRYLIVPGIRSSRIYIVDVK.EPL
WP_132694761.1 IVHQLIMPNRGDEFHHFGWNACSSALSPLTGHAFLERRYLIIPGIRSSRIYVVDVK.EPR
WP_092858204.1 IVHQLLMPHKGDEFHHFGWNACSSSLSPLSGHAFLERRYLIVPGIRSSRIYVIDVK.EPL
WP_040617847.1 IVHQVIMPNTGDEFHHFGWNACSSALSPLSGHAFLERRYLIIPGIRSSRIYVVDVK.EPR
WP_074221017.1 IVHQVIMPNTGDEFHHFGWNACSSALSPLSGHAFLERRYLIIPGIRSSRIYVVDVK.EPR
WP_093249153.1 IVHMLAMPNKGDEFHHFGWNACSSALSPLTGHAFLQRRYLIIPGIRSSRIYVIDTQPDPR
WP_155041248.1 VLSTLAMPHKGDEFHHFGWNACSSSLSPLTDHAFLERRYLIIPGLRSSRIYVVDTKPDPR
WP_200607230.1 IVHKLIMPNKGDEFHHFGWNACSSALSPLSGHAFLERRYLIIPGIRSSRIYVVDVKGGPT
WP_118942321.1 IVHTVVMPNKGDEFHHFGWNACSSALSPLTGHAFLERRYLIIPGIRSSRIYIIDVKGGPK
WP_109423028.1 VVHQVIMPNKGDEFHHFGWNACSSSLSPLSGHAFLERRYLIIPGIRSSRIYVVDVK.EPL
WP_011568565.1 VIHRTEMPVIGDELHHMGWNACSSCNTDGS....MERKYLIIPGVRTTNFYIVDTATDPR
WP_248302375.1 VIHRLEMPGIGDELHHMGWNACSSCHGDAD....MSRQFLILPGVRTTNLHIVDTATNPK
WP_015494695.1 VIHRLEMPGIGDELHHMGWNACSSCHDDAG....MSRKYLLVPGVRSNNIHVIDTATDPY
WP_112323646.1 VIHRLEMPGIGDELHHMGWNACSSCHDDSS....MARKYLLVPGVRSNNIHVIDTATDPR
WP_011048093.1 VIHRLEMPGIGDELHHMGWNACSSCHDDGS....MSRKYLILPGVRSNKLHIVDTATDPR
WP_108386043.1 VIHRLEMPGIGDELHHMGWNACSSCHDDNS....MSRKYLILPGVRSNKLHIVDTATDPR
WP_044050654.1 VIHRLEMPGIGDELHHMGWNACSSCFDDGS....MSRKYLLLPGVRSNNIHVVDTASDPR
WP_049644243.1 VISRLEMPGIGDELHHMGWNACSSCHDDSS....MSRRYLLVPGVRSSNIHVVDTID.PR
*

```
                   120       130       140       150       160       170
EEX10901.1     KAKIHKIIEPEEVFAKTGYSRPHTIHCGPEGIYVSTLGGGGPDGTDGPPGIFIMDCETFD
WP_039535420.1 EAKIHKIIEPEEVFAKTGYSRPHTIHCGPEGIYVSTLGGAGEDGTDGPPGIFIMDCESFE
WP_213890254.1 KAKIHKIIEPEEVFAKTGYSRPHTIHCGPEGIYVSTLGGGGKDGTDGPPGIFIMDCETFD
WP_249712480.1 KAKIHKIIEPEEVFAKTGYSRPHTIHCGPEGIYVSTLGGGGADGTDGPPGIFIMDCESFE
WP_050930027.1 KAKIHKIIEPEEVFEKTGYSRPHTIHCGPEGIYVSTLGGGGKDGTDGPPGIFIMDCETFD
WP_096787502.1 KATIHKIIEPEEVFAKTGYSRPHTIHCGPEGIYVSTLGGGGKDGTDGPPGIFIMDCQTFE
WP_102110101.1 KAKIHRIIEPEELFAKTGYSRPHTIHCGPEGIYVSTLGGGGADGTDGPPGIFIMDCETFD
WP_039687069.1 NAKIHKIIEPEELMAKTGYSRPHTIHCGPEGIYVSTLGGGGEDGTDGPPGIFIMDCESFE
WP_108837869.1 DMKIHKTIEPEELMAKTGYSRPHTIHCGPEGIYVSTLGGGGKDGTDGPPGIFIMDCQTFE
WP_090218864.1 KAKIHKIIEPEELFAKTGYSRPHTIHCGPEGIYVSTLGGGGKDGTDGPPGIFIMDCQTFE
WP_132241184.1 KASIHKIIEPEELFEKTGYSRPHTIHCGPEGIYVSTLGGAGKDGTDGPPGIFIMDCETFE
WP_036173195.1 KAKIHKIIEPEELFARTGYSRPHTIHCGPEGIYVSTLGGGGKDGTDGPPGIFIMDCETFE
WP_011047380.1 KAKIHKIIEPEEVFAKTGYSRPHTIHCGPEGIYVSTLGGGGEDGTDGPPGIFILDCETFE
WP_234172116.1 DAKIHKIIEPEEVFAKTGYSRLHTIHCGPEGIYVSTLGGGGPDGTDGPPGIFIMDCETFD
WP_049642916.1 NAKIHKTIEPEEVFAKTGYSRPHTIHCGPEGIYVSTLGGAGADGTDGPPGIFILDCETFE
WP_147127258.1 KAKIHKIIEPEEVFEKTGYSRPHTIHCGPEGIYVSTLGGGGEDGTDGPPGIFLLDCETFD
WP_218449721.1 KAKIHKIIEPEEVFAKTGYSRPHTIHCGPEGIYVSTLGGGGADGTNGPPGIFILDCESFE
WP_146344021.1 DPKIHKIIEPEEVFAKTGYSRPHTIHCGPEGIYVSTLGGGGVDGTDGPPGIFILDCESFE
WP_012177434.1 KAKIHKIIEPEEVFAKTGYSRPHTIHCGPEGIYVSTLGGGGADGMDGPPGIFIMDCETFE
WP_040650483.1 KAKIHKIIEPEEVFDKTGYSRPHTIHCGPEGIYVSTLGGGGPDGTDGPPGIFILDCETFE
WP_115394491.1 EAKIHKIIEPEEVFAKTGYSRPHTIHCGPEGIYVSTLGGGGPDGTDGPPGIFIMDCETFE
WP_050664078.1 KAKIHKIIEPEEVFAKTGYSRPHTIHCGPEGIYVSTLGGGGKDGTDGPPGMFIMDCETFE
WP_025047812.1 KAHIHKIIEPEELFEKTGYSRPHTIHCGPEGIYVSTLGGGGPDGTDGPPGIFIMDCETFE
WP_015501548.1 KAHIHKTIEPEELFAKTGYSRPHTIHCGPEGIYVSCLGGSGKDGTDGPPGIFIMDCETFE
WP_040169852.1 KAKIHKTIEPEEVFAKTGYSRPHTIHCGPEGIYVSTLGGGGEDGTDGPPGIFIMDCETFE
WP_058311814.1 EAKIHKIIEPEEIFEKTGYSRPHTIHCGPEGIYVSTLGGGGPDGTDGPPGIFIMDCESFE
WP_142083434.1 DLKIHKIIEPEEVFAKTGYSRPHTIHCGPEGIYVSTLGGGGKDGMDGPPGIFIMDCETFD
WP_071690751.1 KASIHKIIEPEEVFAKTGYSRPHTIHCGPEGIYVSTLGGGGEDGTDGPPGIFIMDCETFE
WP_108385718.1 KAKIHKTIEPEEIFAKTGYSRPHTIHCGPEGIYVSTLGGGGPDGTDGPPGIFIMDCETFD
WP_171173290.1 EAKIHKIIEPEEVFAKTGYSRPHTIHCGPEGIYVSTLGGGGADGTDGPPGIFIMDCETFD
WP_132694761.1 APKIHKIIEPEEVFARTGYSRPHTIHCGPEGIYVSTLGGGGPDGTDGPPGIFIMDCETFE
WP_092858204.1 KAKIHKIIEPEEVFARTGYSRPHTIHCGPEGIYVSTLGGGGPEGTDGPPGIFIMDCESFE
WP_040617847.1 KAKIHKIIEPEEVFAKTGYSRPHTIHCGPEGIYVSTLGGGGKDGTDGPPGIFIMDCETFD
WP_074221017.1 KAQIHKIIEPEEIFSKTGYSRPHTIHCGPEGIYVSTLGGGGPDGTDGPPGIFILDCQTFD
WP_093249153.1 KPTIHKIIEPEELFARTGYSRPHTVHCGPEGVYVSTLGGGGADGTDGPPGIFIMDCETFE
WP_155041248.1 HPTIHRVIEPEEVFRKTGYSRPHTIHCGPEGIYVSTLGGGGPDGTDGPPGIFIMDCQTFD
WP_200607230.1 AAKIHRIIEPEEVFAKTGYSRPHTIHCGPEGIYVSTLGGGGKDGTDGPPGIFIMDCETFD
WP_118942321.1 EAKIHKIIEPEELFAKTGYSRPHTIHCGPEGIYVSTLGGGGKDGTDGPPGIFIMDCETFD
WP_109423028.1 KAKIHKIIEPEEVFAKTGYSRPHTIHCGPEGIYVSTLGGGGEDGTDGPPGIFIMDCETFE
WP_011568565.1 KPTLFKTIDGEEIKAKTNLSAPHTVHCLGADIIVSMLG.....DADGNGPGGFLHLDKEFN
WP_248302375.1 APKLHMIVSGDEIKEKTNLSAPHTVHCLGSDIIISMLG.....DADGNGPGGFLHLDKDFN
WP_015494695.1 APRLHKVIDGAEIKSKTNLSGPHTVHCLGSEIIISFLG.....DAQGEAPGGYLQLNKDFE
WP_112323646.1 APRLHKIIDGAEIRAKTDLSGPHTVHCLGSEIIISFLG.....DARGEAPGGYLHLDKDFE
WP_011048093.1 APRLHKVIDGAEIKAKADLSGPHTVHCLGSEIIISFLG.....DAKGEAPGGYLHLNKDFN
WP_108386043.1 APRLHKVIDGAEIKAKADLSGPHTVHCLGSEIIISFLG.....NAKGEAPGGYLHLDKEFN
WP_044050654.1 APRLHKVIDGAEIKSKTNLSGPHTVHCLGSEIIISMLG.....DAKGEAPGGYLHLNKDFD
WP_049644243.1 NPVLHKVIEGTEIKAKTNLSAPHTVHCLGSEIIISMLG.....DAKGEAPGGYLHLNKDFE
                           *
```

```
                 180       190       200       210       220       230
EEX10901.1     IIGRYEIERGVQDKHYDFWWNLPRDYMVSSEWGLPPQFENGIVAEDLLSNKYGHRIHFWN
WP_039535420.1 IIGRYEIERGVQDKHYDFWWNLPRDYMVSSEWGLPPQFENGIVAEDLLSNKYGHRIHFWN
WP_213890254.1 IIGRWEMDRGVQDKHYDFWWNLPRDYMVSSEWGLPPQFENGIVAEDLLSNKYGHSIHFWD
WP_249712480.1 ILGRYEMDRGVQDKHYDFWWNLPRDYMVSSEWGLPPQFENGIVAEDLLSNKYGHSIHFWD
WP_050930027.1 ILGRYEMDRGIQDKHYDFWWNLPRDYMVSSEWGLPPQFENGIVAEDLLSNKYGHKIHFWD
WP_096787502.1 VLGRYEMDRGSQDKHYDFWWNLPRDYMVSSEWALPPQFENGLVPEDLLGNRYGHTIHFWD
WP_102110101.1 ILGRYEMDRGIQDKHYDFWWNLPRDYMVSSEWGLPPQFENGVVPEDLLSNKYGHTIHFWD
WP_039687069.1 ILGRYEMDRGIQDKHYDFWWNLPRDYMVSSEWGLPPQFENGVVPEDLLSNKYGHTIHFWD
WP_108837869.1 IQGRYEMDRGIQDKHYDFWWNLPRDYMVSSEWGLPPQFENGIVAEDLLSNKYGHTIHFWD
WP_090218864.1 ILGRYEMERGIQDKHYDFWWNLPRDYMVSSEWGLPPQFENGIVAEDLLSNKYGHTIHFWD
WP_132241184.1 ILGRYEMDRGAQDKHYDFWWNLPRDYMVSSEWGLPPQFENGIVPEDLLSNKYGHSIHFWD
WP_036173195.1 ILGRYEMDRGAQDKHYDFWWNLPRDYMVSSEWGLPPQFENGVVPEDLLSNRYGHAIHFWD
WP_011047380.1 ILGRYEMDRGEQTKHYDFWWNLPRDYMVSSEWGLPPQFENGIVPEDLLSNKYGHKIHFWK
WP_234172116.1 VIGRYEIDRGPQNKQYDFWWNLPRDYMVSSEWGLPPQFENGIVPEDLLSNKYGHRIHFWD
WP_049642916.1 ILGRYEMDRGVQDKHYDFWWNLPRDYMVSSEWGLPPQFENGLVPEDLLSNKYGHSIHFWD
WP_147127258.1 IIGRYEIDRGTQDKHYDYWWNLPQNYMVSSEWGLPPQFENGVVPEDLLSNKYGHRIHFWD
WP_218449721.1 ILGRYEMDRGKQDKHYDFWWNLPRDYMVSSEWALPPQFENGIVPEDLLSNKYGHSIHFWN
WP_146344021.1 ILGRYEMDRGAQDKHYDFWWNLPRDYMISSEWGLPPQFENGIVAEDLLSNKYGHSIHFWD
WP_012177434.1 ILGRYEMDRGKQDKHYDFWWNLPRDYMVSSEWGLPPQFENGIVAEDLLANKYGHSIHFWN
WP_040650483.1 ILGRYEMDRGQQDKHYDFWWNLPRDYMVSSEWGLPPQFENGLVAEDLLSNKYGHKIHFWK
WP_115394491.1 ILGRYEMDRGKQDKHYDFWWNLPRDYMVSSEWGLPPQFENGIVAEDLLSNKYGHSIHFWN
WP_050664078.1 ILGRYEMDRGVQDKQYDFWWNLPRNYMVSSEWGLPPQYENGIVPEDLLSNKYGHSIHFWD
WP_025047812.1 IIGRYEMDRGAQDKHYDFWWNLPQDYMVSSEWGLPPQYENGVVAEDLLSNKYGHSIHFWD
WP_015501548.1 IIGQYEMDRGKQDKHYDFWWNLPQDYMVSSEWGLPPQFENGVVPEDLMSNKYGHSIHFWD
WP_040169852.1 IIGRYEMDRGKQDKHYDFWWNLPQDYMVSSEWGLPPQFENGVVPEDLLSNKYGHSIHFWD
WP_058311814.1 VIGRYEMDRGPQDKHYDFWWNLPQDYMVSSEWGLPPQFENGIVPEDLLSNKYGHSIHFWN
WP_142083434.1 IIGRYEMDRGKQEKHYDFWWNLPQDYMVSSEWGLPPQFENGLVPEELLSNKYGHALHFWD
WP_071690751.1 IIGQYEMDRGKQDKHYDFWWNLPQDYMVSSEWGLPPQFENGIVAEDLLSNKYGHSIHFWD
WP_108385718.1 IIGQYEMDRGEQDKHYDFWWNLPQDYMVSSEWGLPPQFENGIVAEDLLSNKYGHKIHFWD
WP_171173290.1 IIGQYEMDRGPQDKHYDFWWNLPQDYMVSSEWGLPPQFEGGLVAEDLLSNKYGHSIHFWD
WP_132694761.1 IRGRYEMDRGRQDKHYDFWWNLPRDYMVSSEWGLPPQFENGLVPEDLLSNRYGHAIHFWK
WP_092858204.1 ILGRYEMDRGQQEKHYDFWWNLPRDYMVNSEWGLPPQFENGLVPEDLLSNKYGHRIHFWN
WP_040617847.1 IIGRYEMDRGLQDKHYDFWWNLPRNYMVSSEWGIPPQFENGLVPEDLLSNKYGHSIHFWD
WP_074221017.1 VIGRYEIDRGSQDKHYDFWWNLPRDYMVSSEWGLPPQFENGLVAEDLLSNKYGHRLHFWN
WP_093249153.1 IRGRWEMDRGPQDKHYDFWWNLPRDYMVSSEWGLPPQYENGVVPEDLLANRYGHSLHFWD
WP_155041248.1 VIGRWEIDRGPQDKHYDFWWNLPRDYMVSSEWALPPQFEGGIVPEQLLSNKYGHRLHFWD
WP_200607230.1 VIGRYEIDRGPQDKQYDFWWNLPRDYMVSSEWGLPPQFEGGVVPEDLLSNKYGHRLHFWD
WP_118942321.1 IIGRYEMDRGAQDKHYDFWWNLPRDYMVSSEWGLPPQFENGIVAEDLMGGKYGHKIHFWD
WP_109423028.1 VIGRYEMDRGPQDKHYDFWWNLPRDYMVSSEWGLPPQFENGIVPGDLLSNQYGHRIHFWD
WP_011568565.1 IIGRWENDLGDMKYNYDFWYQPRHNMMISTEWGSPNTFMPGFDLEDVARGKYGQHVHFWD
WP_248302375.1 IVGRWENDLGDMKYNYDFWYQPRHNTMVSSEWGSPNTFMPGFDLGDVAKGKYGQQIHFWN
WP_015494695.1 IVGRWEETMGDIPFSYDFWYQPRHNVMVSSEWAAPNTFMPGFDLEEVGHLKYGRRLHIWD
WP_112323646.1 IVGRWEASMGDIPFGYDFWYQPRHNVMISSEWAAPNTFMPGFDLEEVGYLKYGRRLHVWD
WP_011048093.1 IVGRWENSMGDIKFGYDFWYQPRHNVMVSSEWAAPNTFMPGFDLEEVGHLKYGRELHFWD
WP_108386043.1 IVGRWENSMGDIKFGYDFWYQPRHNVMVSSEWAAPNTFMPGFDLEEVGHLKYGRELHFWD
WP_044050654.1 IVGRWENSMGDIKFGYDFWYQPRHNVMVSSEWAAPNTFMPGFDLEEVGHLKYGREIHFWD
WP_049644243.1 IVGRWESSMGDIKFGYDFWYQPRHNVMVSSEWAAPNTFMPGFDLEEVGHLKYGREIHFWD
```

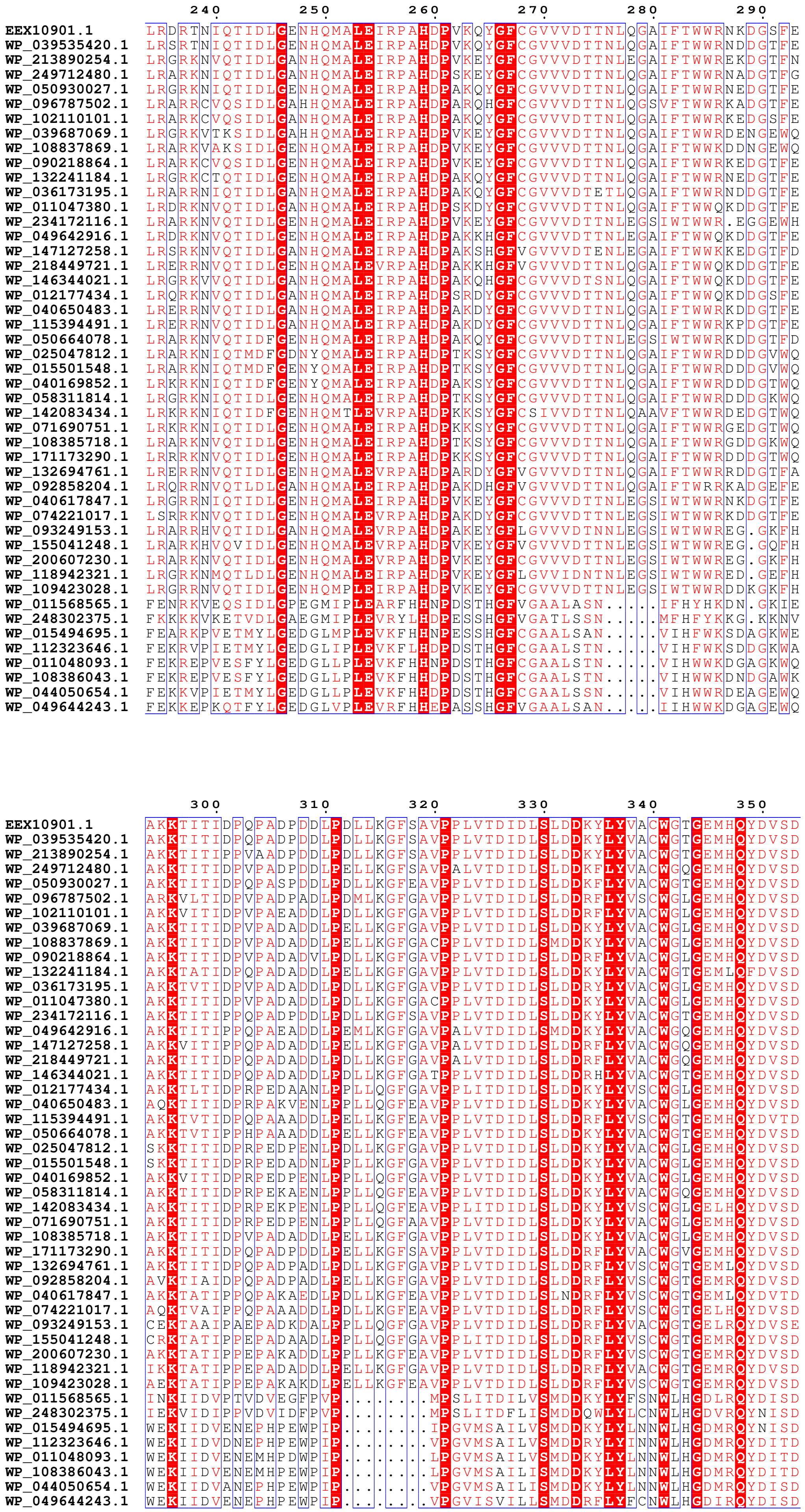

240 250 260 270 280 290
EEX10901.1
WP_039535420.1
WP_213890254.1
WP_249712480.1
WP_050930027.1
WP_096787502.1
WP_102110101.1
WP_039687069.1
WP_108837869.1
WP_090218864.1
WP_132241184.1
WP_036173195.1
WP_011047380.1
WP_234172116.1
WP_049642916.1
WP_147127258.1
WP_218449721.1
WP_146344021.1
WP_012177434.1
WP_040650483.1
WP_115394491.1
WP_050664078.1
WP_025047812.1
WP_015501548.1
WP_040169852.1
WP_058311814.1
WP_142083434.1
WP_071690751.1
WP_108385718.1
WP_171173290.1
WP_132694761.1
WP_092858204.1
WP_040617847.1
WP_074221017.1
WP_093249153.1
WP_155041248.1
WP_200607230.1
WP_118942321.1
WP_109423028.1
WP_011568565.1
WP_248302375.1
WP_015494695.1
WP_112323646.1
WP_011048093.1
WP_108386043.1
WP_044050654.1
WP_049644243.1
300 310 320 330 340 350

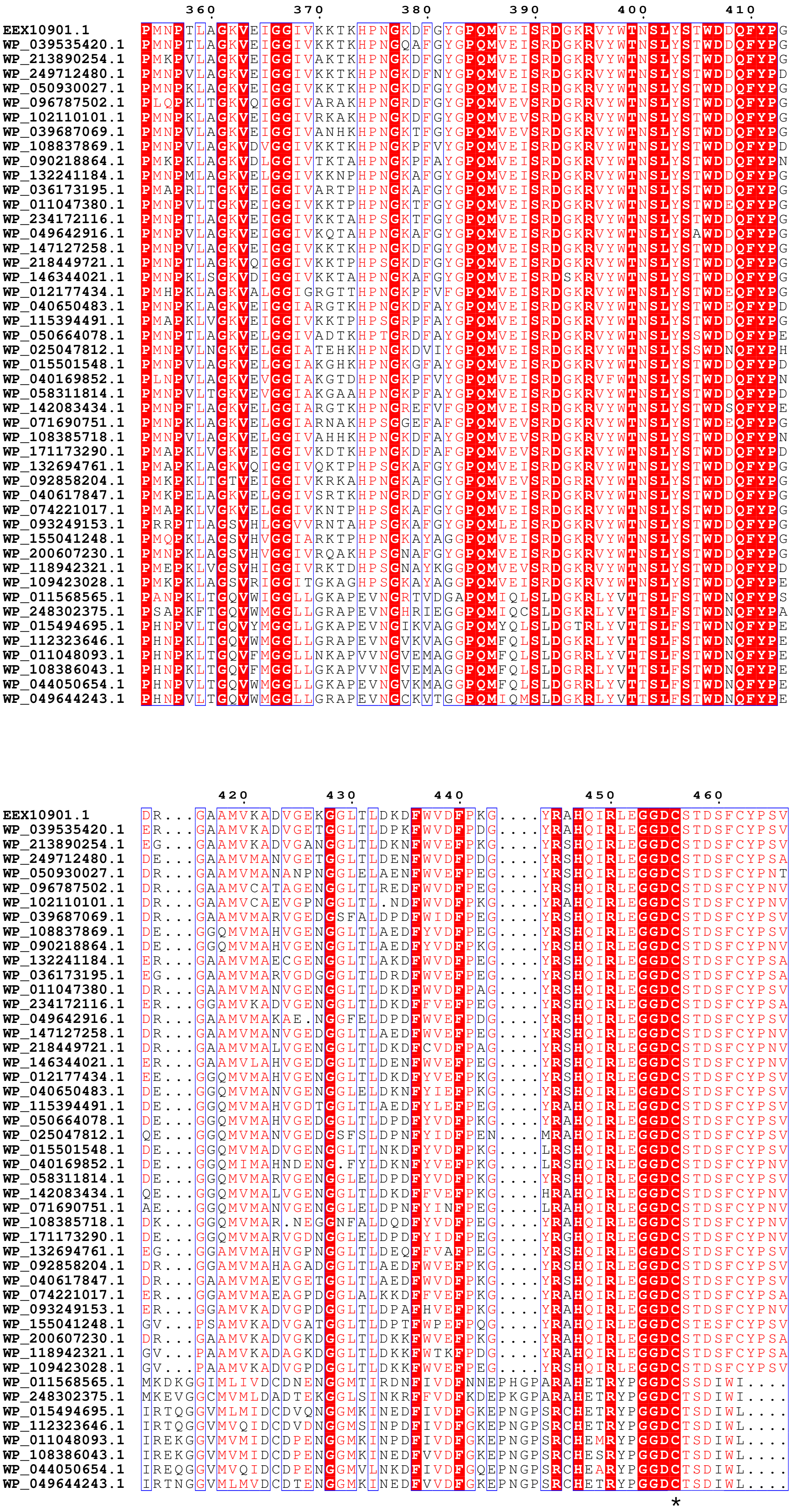
360
370
380
390
400
410
EEX10901.1
WP_039535420.1
WP_213890254.1
WP_249712480.1
WP_050930027.1
WP_096787502.1
WP_102110101.1
WP_039687069.1
WP_108837869.1
WP_090218864.1
WP_132241184.1
WP_036173195.1
WP_011047380.1
WP_234172116.1
WP_049642916.1
WP_147127258.1
WP_218449721.1
WP_146344021.1
WP_012177434.1
WP_040650483.1
WP_115394491.1
WP_050664078.1
WP_025047812.1
WP_015501548.1
WP_040169852.1
WP_058311814.1
WP_142083434.1
WP_071690751.1
WP_108385718.1
WP_171173290.1
WP_132694761.1
WP_092858204.1
WP_040617847.1
WP_074221017.1
WP_093249153.1
WP_155041248.1
WP_200607230.1
WP_118942321.1
WP_109423028.1
WP_011568565.1
WP_248302375.1
WP_015494695.1
WP_112323646.1
WP_011048093.1
WP_108386043.1
WP_044050654.1
WP_049644243.1
420
430
440
450
460
*

```
EEX10901.1      .
WP_039535420.1  .
WP_213890254.1  .
WP_249712480.1  .
WP_050930027.1  .
WP_096787502.1  .
WP_102110101.1  .
WP_039687069.1  G
WP_108837869.1  .
WP_090218864.1  .
WP_132241184.1  .
WP_036173195.1  .
WP_011047380.1  .
WP_234172116.1  .
WP_049642916.1  .
WP_147127258.1  .
WP_218449721.1  .
WP_146344021.1  .
WP_012177434.1  .
WP_040650483.1  .
WP_115394491.1  .
WP_050664078.1  .
WP_025047812.1  .
WP_015501548.1  .
WP_040169852.1  .
WP_058311814.1  .
WP_142083434.1  .
WP_071690751.1  .
WP_108385718.1  .
WP_171173290.1  .
WP_132694761.1  .
WP_092858204.1  .
WP_040617847.1  .
WP_074221017.1  .
WP_093249153.1  .
WP_155041248.1  .
WP_200607230.1  .
WP_118942321.1  .
WP_109423028.1  .
WP_011568565.1  .
WP_248302375.1  .
WP_015494695.1  .
WP_112323646.1  .
WP_011048093.1  .
WP_108386043.1  .
WP_044050654.1  .
WP_049644243.1  .
```

Figure S10. Amino acid sequence alignment of 49 cluster 1 MTOs. Conserved cysteine residues are marked with asterisk (*).

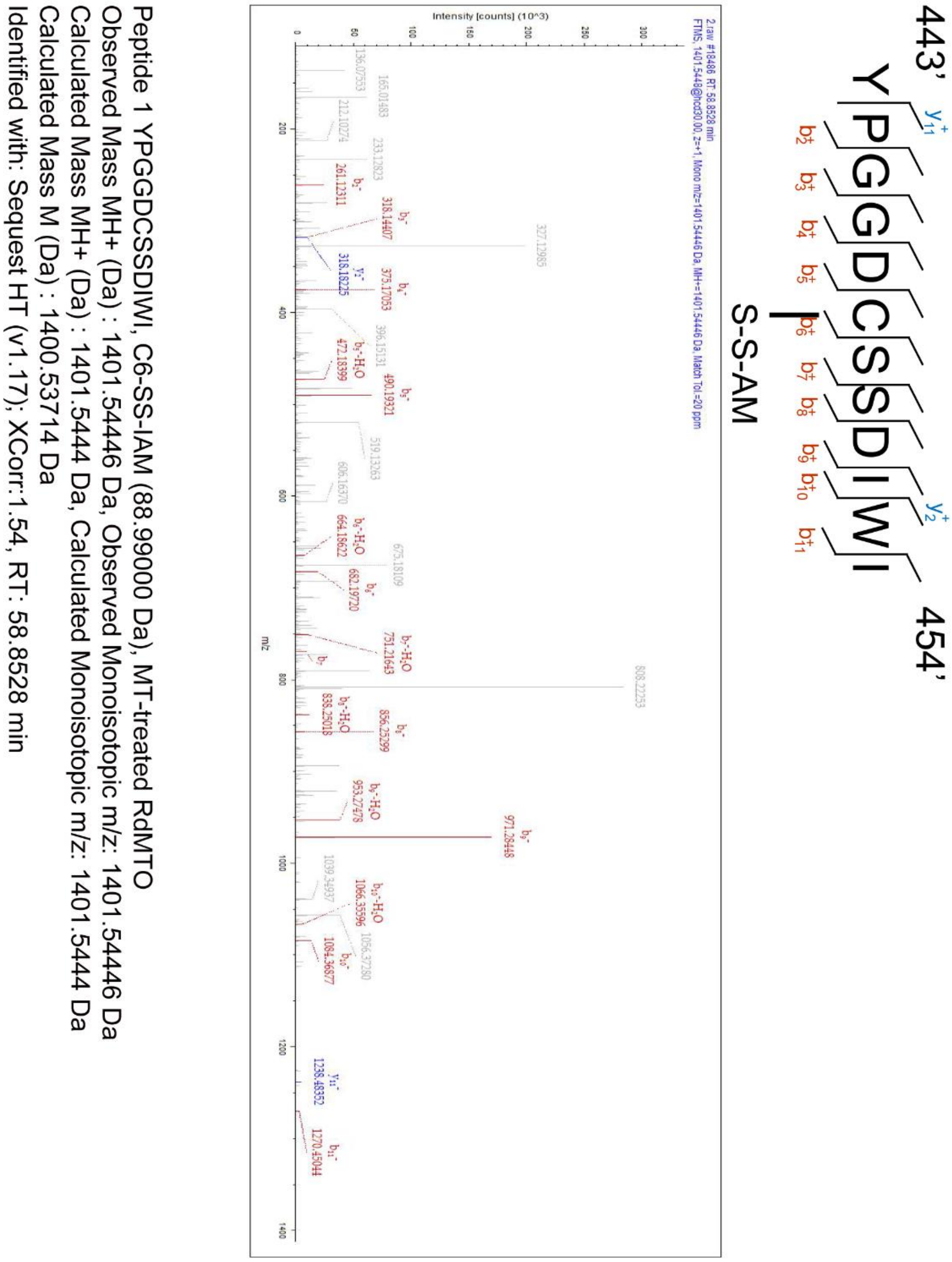

Figure S11. MS^2 spectra of peptide 1 (Cys448-S-S-AM) (from MT-treated RdMTO).

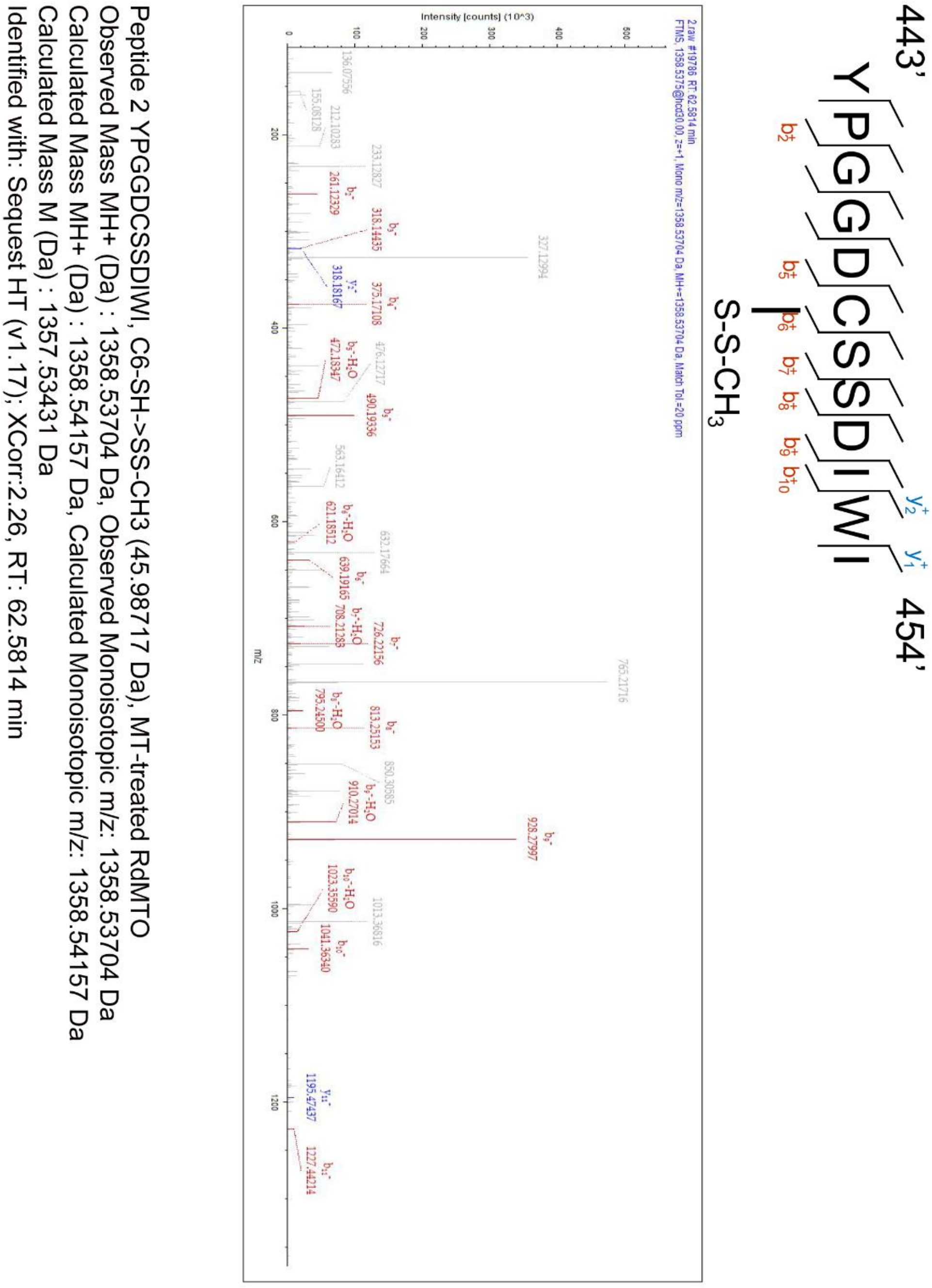

Figure S12. MS^2 spectra of peptide 2 (Cys448-SS-CH_3) (from MT-treated RdMTO).

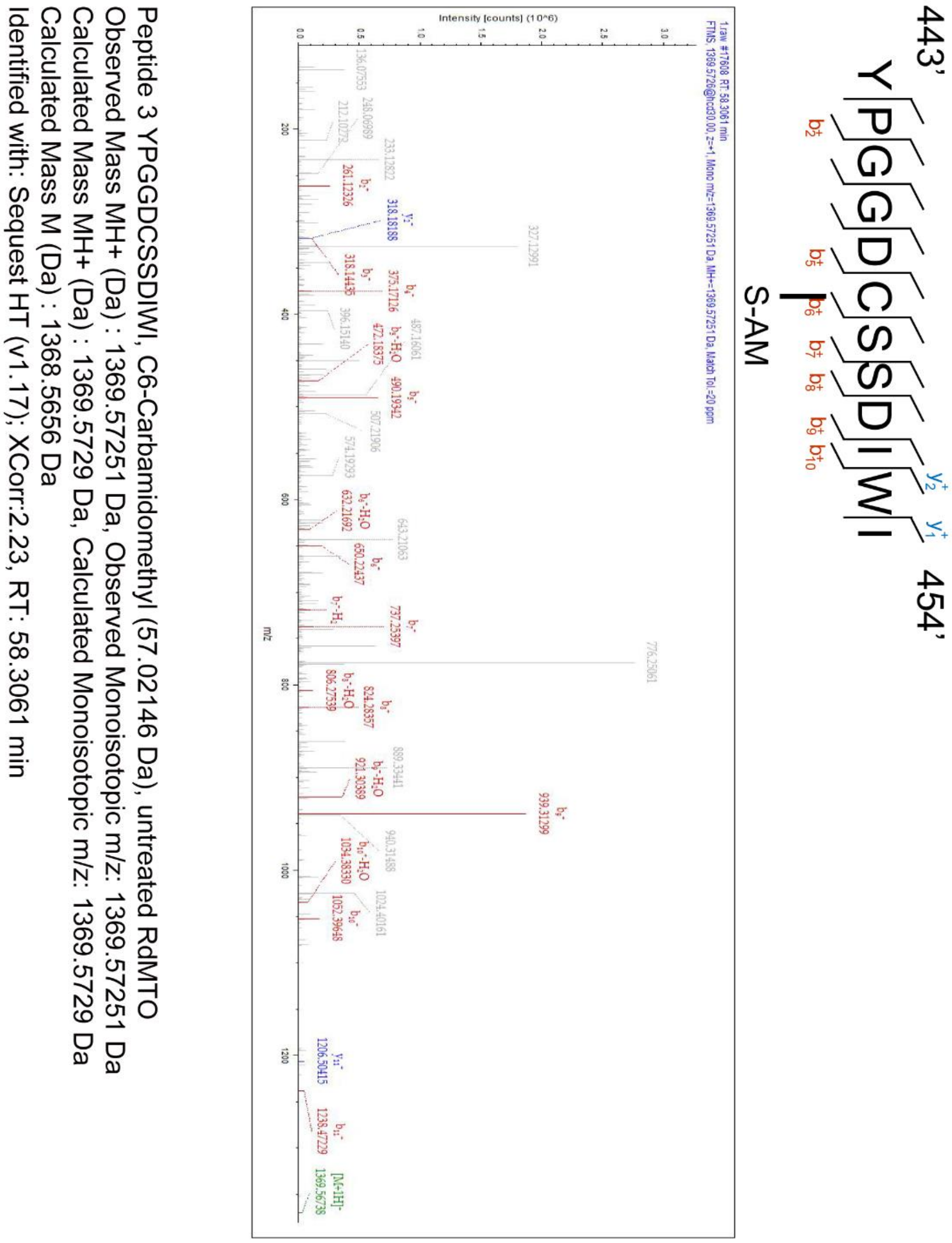

Figure S13. MS^2 data of peptide 3 (Cys448-S-AM) (from untreated RdMTO).

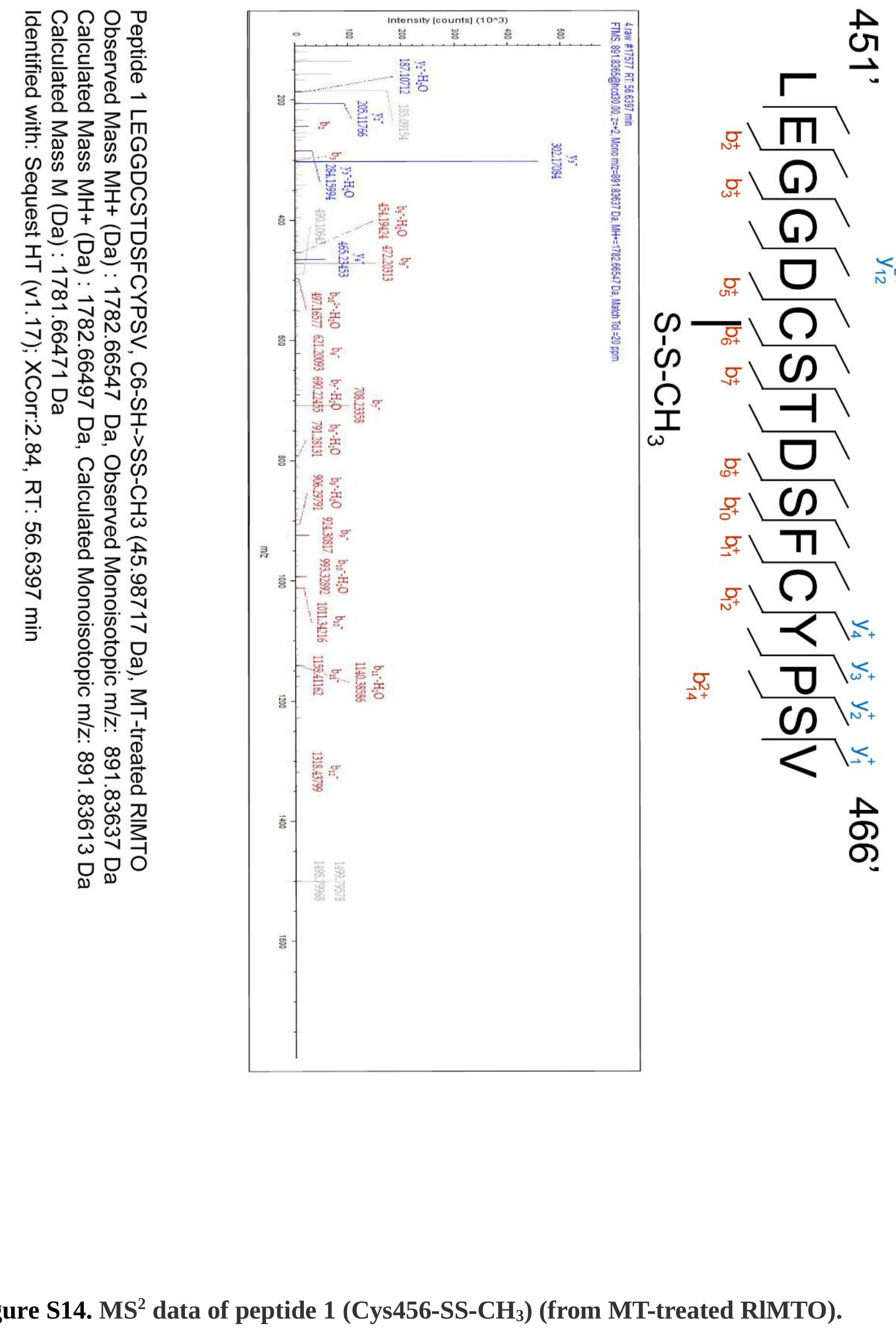

Figure S14. MS^2 data of peptide 1 (Cys456-SS-CH$_3$) (from MT-treated RlMTO).

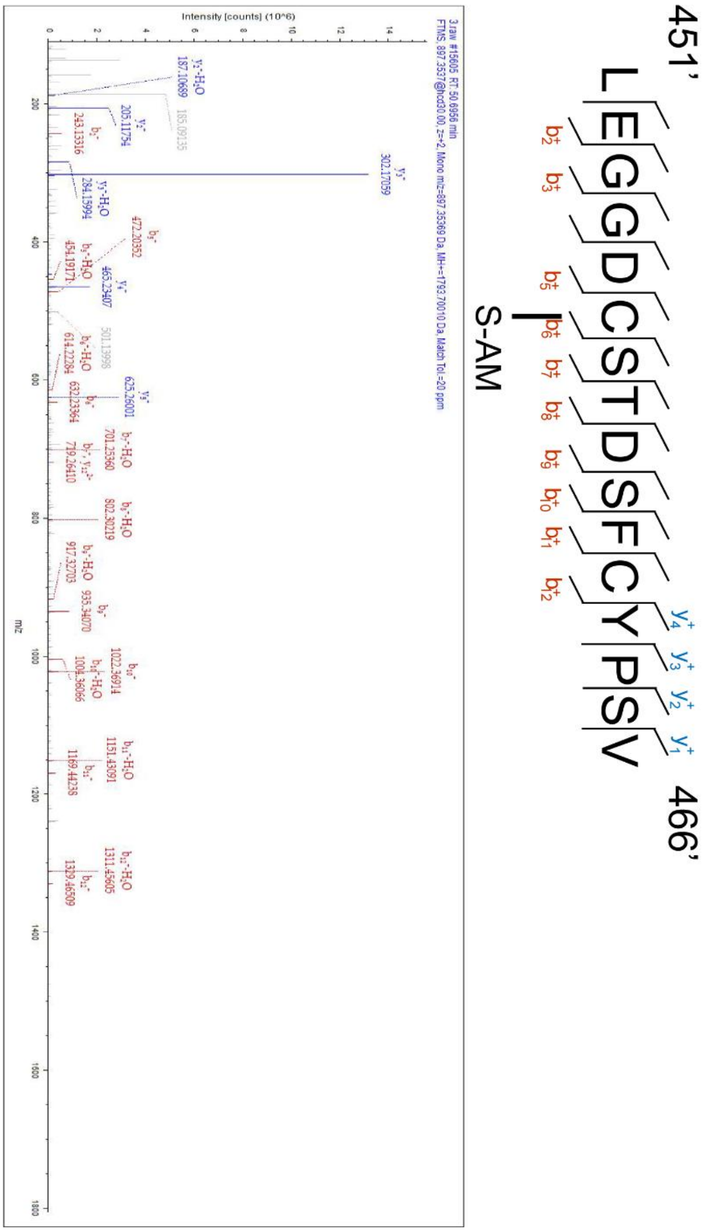

Peptide 2 LEGGDCSTDSFCYPSV, C6-Carbamidomethyl (57.02146 Da), untreated RlMTO
Observed Mass MH+ (Da) : 1793.70010 Da, Observed Monoisotopic m/z: 897.35369 Da
Calculated Mass MH+ (Da) : 1793.6993 Da, Calculated Monoisotopic m/z: 897.35329 Da
Calculated Mass M (Da) : 1792.6920 Da
Identified with: Sequest HT (v1.17); XCorr:2.86, RT: 50.6956 min

Figure S15. **MS^2 data of peptide 2 (Cys456-S-AM) (from untreated RlMTO).**

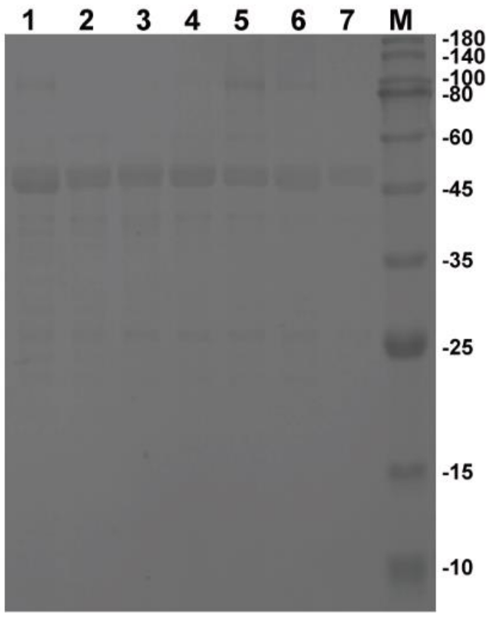

Figure S16. Non-Reduced SDS-PAGE analysis of the mutant RdMTO. M, molecular weight marker (weights in kDa); 1. RdMTO$_{C82S}$ mutant; 2. RdMTO$_{C143S}$ mutant; 3. RdMTO$_{C143SC448S}$ mutant; 4. RdMTO$_{C82SC143S}$ mutant; 5. RdMTO$_{C82SC448S}$ mutant; 6. RdMTO$_{C82SC448S}$ mutant; 7. RdMTO$_{C82SC143SC448S}$ mutant.

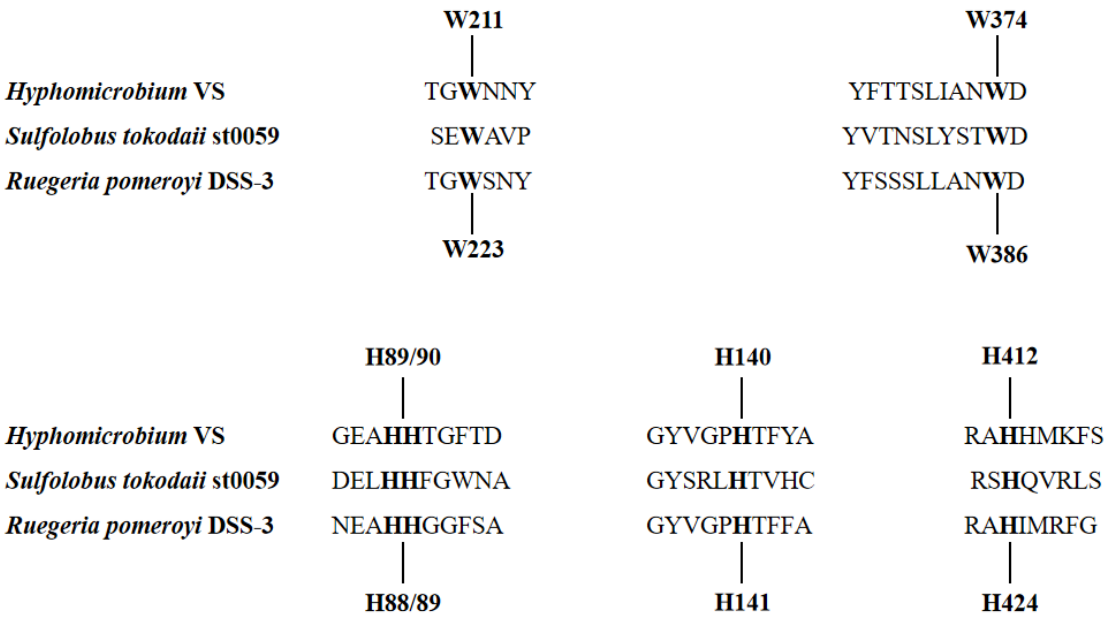

Figure S17. Conservation of the W residues potentially involved in formation of a TTQ cofactor by MauG and putative copper ligands in RpMTO of *Ruegeria pomeroyi* DSS-3 and homologues in *Hyphomicrobium* sp. VS and S*ulfolobus tokodaii*.

REFERENCES

1. Xin YF, Liu HL, Cui FF, Liu HW, Xun LY. 2016. Recombinant Escherichia coli with sulfide: quinone oxidoreductase and persulfide dioxygenase rapidly oxidises sulfide to sulfite and thiosulfate via a new pathway. Environmental Microbiology 18:5123-5136.
2. Chen HQ, Zhou CL, Wang L, Chen JG, Ling B, Fu J. 2011. Terbium (III) chelate complexes as fluorescence energy transfer donor in the determination of formaldehyde in aqueous solutions. Spectrochimica Acta Part a-Molecular and Biomolecular Spectroscopy 78:371-374.
3. Schafer A, Tauch A, Jager W, Kalinowski J, Thierbach G, Puhler A. 1994. Small Mobilizable Multipurpose Cloning Vectors Derived from the Escherichia-Coli Plasmids Pk18 and Pk19 - Selection of Defined Deletions in the Chromosome of Corynebacterium-Glutamicum. Gene 145:69-73.
4. Harighi B. 2009. Genetic evidence for CheB- and CheR-dependent chemotaxis system in A. tumefaciens toward acetosyringone. Microbiological Research 164:634-641.
